# Holistic Monte-Carlo optical modelling of biological imaging

**DOI:** 10.1038/s41598-019-51850-1

**Published:** 2019-11-01

**Authors:** Guillem Carles, Paul Zammit, Andrew R. Harvey

**Affiliations:** School of Physics and Astronomy, University of Glasgow, Glasgow, G12 8QQ UK

**Keywords:** Imaging and sensing, Microscopy

## Abstract

The invention and advancement of biological microscopy depends critically on an ability to accurately simulate imaging of complex biological structures embedded within complex scattering media. Unfortunately no technique exists for rigorous simulation of the complete imaging process, including the source, instrument, sample and detector. Monte-Carlo modelling is the gold standard for the modelling of light propagation in tissue, but is somewhat laborious to implement and does not incorporate the rejection of scattered light by the microscope. On the other hand microscopes may be rigorously and rapidly modelled using commercial ray-tracing software, but excluding the interaction with the biological sample. We report a hybrid Monte-Carlo optical ray-tracing technique for modelling of complete imaging systems of arbitrary complexity. We make the software available to enable user-friendly and rigorous virtual prototyping of biological microscopy of arbitrary complexity involving light scattering, fluorescence, polarised light propagation, diffraction and coherence. Examples are presented for the modelling and optimisation of representative imaging of neural cells using light-sheet and micro-endoscopic fluorescence microscopy and imaging of retinal vasculature using confocal and non-confocal scanning-laser ophthalmoscopes.

## Introduction

Light scattering in biological tissue is the major barrier to imaging biological structures and processes deep within tissue. Techniques such as optical-coherence tomography and confocal, light-sheet or two-photon microscopy improve image contrast by rejection of scattered light^1^, and optimisation of such techniques is critically dependent on a rigorous quantitative understanding of how scattered and unscattered light contribute to recorded images. Conventional numerical modelling of biological microscopy treats light propagation in the biological sample and modelling of the imaging instrument separately and so cannot accurately model their mutual interaction. For example, it is not possible to accurately model a routine and fundamental process, such as imaging of a neuron or vasculature, using a confocal microscope or multi-photon microscope. This inability to model a complete imaging and sensing process contrasts with the field of nuclear and particle physics, where Monte-Carlo simulation tools, such as *Geant*, are able to model particle transmission and scattering within complex targets and instruments to enable quantitative virtual prototyping of complete radiation detection systems^2^. Notably, an add-on to *Geant* also enables Monte-Carlo modelling of the diffuse propagation of radiation-induced light in tissue^3^. We describe here holistic Monte-Carlo optical modelling (HMCOM), for system-level virtual prototyping of biological optical microscopy. We report the first rigorous and holistic modelling of the complete imaging process in scattering media: that is light propagation is modelled in the imaging regime, where polarisation and non-diffuse light propagation is important in addition to interaction of all light with the optical instrument. Holistic modelling incorporates the coherent propagation of polarised light through the complete image-formation process: from source, through the instrument illumination optics, through the biological sample, through the imaging instrument, to the detector. It thus enables rigorous modelling of image formation in turbid media, including the combined effects of light scatter by tissue and instrument optics. Such holistic modelling enables accurate prediction, optimisation and construction of accurate forward-imaging models, and also the generation of ground-truth data for the training of algorithms.

Monte-Carlo techniques are the gold standard for modelling photon transport in biological tissue since they provide high accuracy and can handle realistic and arbitrary 3D structures^4–6^. On the other hand, optical ray tracing is universally used for the design and optimisation of optical instruments. Until now, modelling of image formation within biological media has employed a somewhat disconnected approach: light propagation in tissue and the imaging performance of the instrument are modelled independently and coupled only through high-level characterisations such as tissue and instrument point-spread functions. Light is not propagated coherently between instrument and sample and there is therefore no 'memory’ in the modelling of light propagated between instrument and sample: that is, there is no tracking of light direction, polarisation and phase propagated from source to detector. The physical principles of Monte-Carlo modelling are, however, essentially equivalent to the principles of ray tracing in that both propagate light rays (termed photon packets in the Monte-Carlo literature) between interactions with media. For optical ray tracing, ray lengths, amplitudes and directions are deterministic and calculated according to the laws of physical optics, whereas for Monte-Carlo modelling they are determined stochastically by mean-free paths and angular probability density functions. We exploit this equivalence to integrate ray tracing of optical instruments with Monte-Carlo modelling of light propagation in scattering media within a single hybrid framework.

Polarisation has a profound influence on image formation in scattering media, but existing Monte-Carlo modelling software normally neglects the polarisation state of light. Multiple scattering rapidly depolarises light^7,8^, but significant directional, coherence, and polarisation information is retained by scattered light in the near-ballistic and snake-like regimes^9,10^, important to all biological imaging^11–16^. Accurate modelling of image formation therefore requires that polarisation is tracked, particularly imaging using coherent or partially-coherent light, such as for optical coherence tomography and emerging techniques based on phase conjugation and speckle^17–22^. A small number of previous works have reported Monte-Carlo software that tracks polarisation^23–29^; however, Monte-Carlo modelling of photon diffusion is implemented almost exclusively by user-customised computer code that is not readily customisable to specific biological samples and is therefore, in practice, restricted to simple geometrical shapes such as slabs or spheres. Importantly, Monte-Carlo models do not include the optical instrument in the overall mathematical model and so cannot model the effects of, for example, optical aberrations, depth of field or pupil-engineered masks in a confocal or light-sheet microscope^30–32^.

Algorithms for traditional optical design are well established and invariably employ an approach where optical elements are defined as an ordered sequence of surfaces with specified properties such as curvature and optical properties (first and last surfaces are the light source and image plane respectively). System optimisation involves tracing rays sequentially to calculate optical performance at the image plane. In our implementation of HMCOM we use a modification to this process, where optical components, including the sample, are based on 3D objects, sources and detectors that are freely distributed in space. This defines a framework for Monte-Carlo modelling of complete systems that can readily incorporate optical components with exact prescriptions, 3D models for biological structures and physical-optics models for scattering and fluorescence within turbid media. Complete models can incorporate illumination optics, scattering media, sample and imaging optics (including arbitrary optical components, mirrors, apertures, coatings, 3D structures, etc.), to enable the rigorous modelling of arbitrary microscopy modalities for imaging arbitrary biological structures. Holistic Monte-Carlo optical modelling thus enables system-level, polarimetric and coherent modelling of image formation involving turbid media. We have implemented an accurate polarimetric Mie-scattering model that is also able to simulate fluorescence (see Section “Methods”, and provide a plugin to the optical design software *Zemax-OpticStudio* (Supplementary Software 1). We also demonstrate modelling of diffraction, essential for modelling high-resolution microscopy, using sampling of the angular spectrum of a ray-traced beam to compute its diffracted propagation within an HMCOM model (see Section “Methods” and Supplementary Software 2).

## Results

Validation of our technique is demonstrated by modelling of two exemplar optical configurations: the calculation of the Mueller matrices of optical-backscatter images from an *in silico* turbid slab, and the polarimetric imaging of a complex artificial volume-scattering object which also highlights the importance of polarisation for accurate modelling of imaging in turbid media; see Supplementary Material, Sections 2–3.

We present three illustrative HMCOM simulations of imaging of biological structures: fluorescence imaging of a neuron, labelled with green-fluorescent protein (GFP), embedded in a scattering medium using a graded-index microendoscope; volumetric imaging and reconstruction of the same neuron using a diffraction-limited light-sheet fluorescence microscope; and imaging of retinal vasculature using a scanning laser ophthalmoscope (SLO).

**Figure 1:**
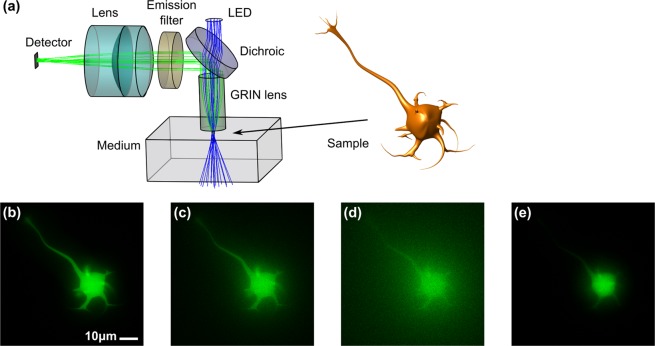
Simulation of fluorescence microscopy of a neuron using a GRIN-lens microendoscope objective. 488 nm illumination rays (in blue) are launched from a LED to approximately uniformly illuminate the sample plane through scattering medium. Fluorescence with a peak wavelength of 510 nm is emitted omnidirectionally and a fraction of the rays (in green) enter the aperture of the GRIN lens and are imaged by the tube lens onto the detector. Images for weak, mild and strong scattering (scattering coefficients *μ*_*s*_ = 0.5, 5 and 10 respectively) are shown in (**b**–**d**) for high chromophore absorbance (i.e. fluorescence occurs close to the sample’s surface) and in (**e**) for weak scattering and low chromophore absorbance (fluorescence is uniform over the sample volume).

The simulation of imaging of a GFP-labelled neuron using a miniature microscope based on a GRIN-lens microendoscope objective is shown in Fig. 1. Rays are propagated using deterministic ray tracing through the microscope optics: from the spatially-extended LED light source, dichroic mirror and the microendoscope into the scattering medium where propagation is calculated using Monte-Carlo methods. A neuron, implemented as a 3D CAD model filled with a specific concentration of GFP is immersed into the scattering medium, and rays entering the neuron stochastically excite fluorescence enabling a quantified calculation of fluorescence radiance. A fraction of the emitted fluorescence rays are captured by the microendoscope aperture and propagate back through the microscope assembly (reflected at the dichroic, through an emission filter, and focused by a doublet tube lens) to form an image at the detector. As can be seen from Fig. 1(b–e), image resolution and contrast is degraded as the scattering coefficient of the tissue increases, as expected. The impact of optical aberrations of the microscope optics is also apparent, in particular the limited depth of field (that is defocus), which blurs the dendritic structures away from the focal plane. Lower levels of other imaging aberrations, such as chromatic aberration, astigmatism, and coma are also present but less apparent in these scattering-degraded images.

**Figure 2:**
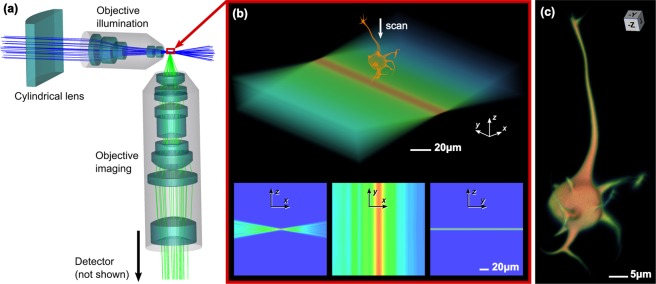
Simulation of a Light-Sheet Fluorescence Microscope. The optical setup including a cylindrical lens and objectives for illumination (12.8x/0.25NA) and imaging (20x/0.4NA) is shown in (**a**). The light-sheet excitation illumination employs monochromatic light at a wavelength of 488 nm (blue rays), and broadband fluorescence emission (green rays) is imaged onto the sensor (not shown). A 3D diffraction pattern is computed from the illumination rays using propagation of the angular spectrum, and is shown in (**b**). Fluorescence rays are generated in the illuminated parts of the sample volume as it is scanned through the light sheet, generating image slices that yield the 3D reconstruction in (**c**) and Supplementary Video 1. Scattering and absorption in the medium and in the sample can be included, see Fig. S4 and Supplementary Video 2.

The second example is the simulation of 3D imaging of the same neuron using a light-sheet fluorescence microscope, as shown in Fig. 2. This traditional system comprises laser excitation, a cylindrical lens and a microscope objective to form a light-sheet at the sample plane, and an orthogonal objective to image fluorescence from within the light sheet onto a detector. The illumination light sheet, including absorption and diffraction within the embedding medium, is shown in Fig. 2(b). Weak scattering within the embedding medium and sample-induced absorption and refraction may also be simulated (see Section “Simulation of scattering in diffracted beams” below, and Supplementary Fig. 4). Translation of the fluorescent sample produces a 3D image of the cell; see Fig. 2(c) and Supplementary Videos 1 and 2. This example demonstrates the integration of ray tracing through the optical system with diffraction (in this case for calculating the light sheet excitation) to model fluorescence within the sample. Newer techniques, using aberration-limited Airy-beam light sheets for example^30,33^, may thus be readily modelled.

For the simulations in both Figs 1 and 2 monochromatic illumination at wavelength *λ* = 488 nm was used, and four wavelengths were defined to sample the broadband fluorescence emission. Weighted summation of the images recorded at the sampled wavelengths provides a good approximation to images recorded with broadband spectra, including the spectral effects of the sample and, for example, dispersion and spectral selectivity in the optics. Selected wavelengths with representative spectra for a dichroic mirror, emission filter and excitation/emission curves for EGFP are shown in Fig. S1.

**Figure 3:**
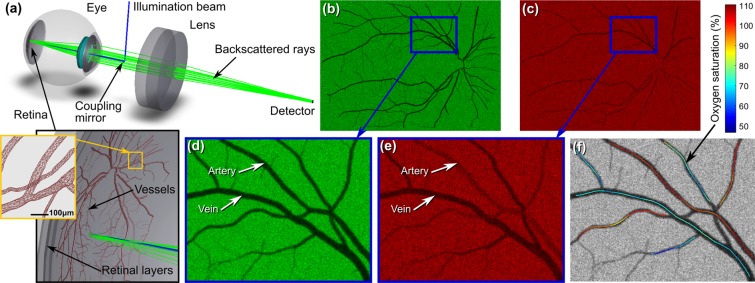
Simulation of retinal imaging and vasculature contrast in a SLO. The setup in (**a**) comprises a coupling mirror that directs illumination rays towards the retina (in blue), where they are scattered and a fraction (in green) are transmitted back through the eye pupil and focused by the lens onto a detector. The close-up image in (**a**) shows the retinal layers that include blood vessels with an example of backscattered rays (only those transmitted by the pupil are shown). Angular raster scanning of the beam yields the retinal images shown in (**b,d**) for 532 nm and in (**c,e**) for 633 nm. Vascular contrast is determined by the interaction of the pinhole and the multiple light paths due to scattering and light absorption by blood in the vessels. The differential contrast of arteries and veins is due to oxygenation-sensitivity of the blood absorption spectrum. Computation of absorption from images (**d,e**) enables application of two-wavelength oximetry shown in (**f**); see Supplementary Figs 9, 10 and Section 5 of Supplementary Material.

The third simulation is of image formation in a scanning laser ophthalmoscope (SLO), as shown in Fig. 3. An illumination beam is focused by the eye to a diffraction-limited spot at the retina. Angular scanning sweeps the spot across the retina and the light scattered back through the pupil is recorded by the SLO to yield an image. Vascular contrast arises from light absorption by haemoglobin and the variation in extinction coefficient with wavelength and oxygen saturation yields dissimilar contrast for arteries and veins. Simulated images and oximetry of networks of retinal arteries and veins recorded with this virtual-prototype SLO are shown in Fig. 3. Such system-level virtual prototyping enables quantification and understanding of the intricate interactions of light with complex retinal structures and pigmentation and how their interaction with the optics of the instrument affect images, to support accurate optimisation of instrument parameters such as confocality and quantitative assessment of functions such as vascular oximetry (see Supplementary Figs 5, 6, 9, 10 and Section 5 of Supplementary Material).

The eye was simulated within HMCOM using a schematic model of a human eye, to which we added a 3D CAD network of vasculature embedded within the retina (Supplementary Material, Section 4). We employed Monte-Carlo modelling (within HMCOM) to simulate the scattering of the illumination spot into a 3D volume of the retina. A fraction of this scattered light exits from this volume towards the anterior of the eye and a small fraction is transmitted through the pupil, undergoing focusing (collimation) by the eye lens and cornea, and is then focused by the SLO onto an image of the illuminated volume at a pixelated detector. The summation of the detector pixels can be configured to implement various modalities of SLO. Confocal detection is simulated using a small pinhole, bucket (that is, non-confocal) detection is simulated by summing a larger disc of pixels and hybrid schemes involving arbitrary compound pinholes^32,34–36^ can also be easily and accurately modelled. While a large bucket detector yields the highest signal at the detector, reduction of the detector (pinhole) diameter towards confocality provides a useful increase in vascular contrast^37^, which is also particularly beneficial for vascular oximetry^32^, but with a reduced signal power.

The contrast of blood vessels is dominantly due to the absorption of light by haemoglobin. Sensitivity of the haemoglobin absorption spectrum to oxygen saturation enables vascular oximetry using the measured contrast of vessels at two or more wavelengths using a simplified optical model for optical absorption based on the Beer-Lambert law as described in Section 5 of Supplementary Material. The uncertainty in oximetry due to noise-induced uncertainty in measured vascular contrast is lowest when the vascular contrast is close to 46% (corresponding to an optical density of log_10_(1/*e*)) and improves with increasing signal-to-noise ratio. Inference of oxygen saturation requires however knowledge of optical path lengths through the blood, an understanding of the effects of scatter and how they interact with the imaging system^38,39^. Typically a simplified model is employed: light detected using confocal imaging is considered to have been transmitted twice through a blood vessel (the dominant reflection is normally considered to be scattering by the choroid or the sclera), whereas for imaging using a large-area detector, light is considered to be transmitted only once through a vessel. It is then scattered into the surrounding retinal-tissue volume, and the light emitted from this volume is imaged by the detector. More generally, the relative weightings of single-pass and double-pass light that contribute to recorded images, are obtained by fitting approximate models to recorded data and extrapolated from a training set of retinas to images recorded of test retinas. Even following careful calibration^40,41^ this model leads to high uncertainty in oximetry. Such approximate models do not provide a route to absolute and reliable assessment of oxygenation or to system optimisation. By way of example, we describe here the use of HMCOM for quantitative virtual prototyping of a SLO to enable its optimisation for vascular contrast and also for improved oximetry in the presence of additional variables, such as local pigmentation and retinal structure.

Using our HMCOM virtual prototype of the SLO we modelled how the precision of oximetry varies with pinhole diameter (trading the higher optical throughput of a larger pinhole against the benefit of the higher contrast of a smaller pinhole). In our model this yields a minimum standard deviation of repeated oximetry of the larger veins (venular oxygenation has greater clinical importance than arterial oxygenation) for a pinhole diameter of 80–120 μm as summarised in Fig. S12. It should be noted that retinal parameters such as pigmentation, vascular caliber, retinal structure, hematocrit, etc., which all affect vascular contrast, vary both between eyes and within eyes and so improved system optimisation and oximetric inversion requires modelling of a representative range of retinas. Construction of an eye-specific forward HMCOM model of vascular also provides a route to improved inversion.

Note that oximetry is just one, albeit prominent, example of a need for quantitative modelling and measurement in the retina: the ability to quantify other chromophores is also important, including macular pigment, melanin, lipofuscin and the visual pigments found in the photoreceptors. Virtual prototyping of instruments to image and quantify such chromophores is a vital tool for developing future ophthalmic instruments.

Detailed description and further discussion on the examples summarised in Figs 1–3 are presented in Supplementart Material, Section 1, and the simulation files with example code are provided in Supplementary File 1.

In summary, recognising the equivalence between the ubiquitous Monte-Carlo methods and optical ray tracing we have implemented the first tool that is able to provide system-level holistic modelling of imaging within turbid media. The inclusion of full polarisation tracking enables rigorous modelling of modern imaging techniques that employ propagation of polarised and/or coherent light through biological samples. Implementation using commercial ray-tracing software enables, for the first time, rapid, rigorous and user-friendly virtual prototyping, including realistic optical components (employing exact optical prescriptions, which can be downloaded from vendors for example) and biological structures of arbitrary complexity (imported from CAD models for example; in this paper we start from the assumption that models are available, nonetheless an example of building a volumetric model of the vasculature for the simulation in Fig. 3 is described in Supplementary Material, Section 4). Importantly, complete and realistic optical models can be rapidly constructed by a non-expert, whereas current far less rigorous approaches typically require dedication of highly-skilled experts to implement. It is noteworthy that HMCOM enables rigorous analysis, system optimisation and construction of realistic forward imaging models, which can aid computational recovery of images from noisy data or system inversion. Importantly, it provides a mechanism for generation of image data to fuel the training of data-starved machine-learning techniques for superior image reconstruction and information extraction^42^. Although our emphasis here is on biological imaging, the tool is equally pertinent to optical sensing with a range of turbid media, such as foams, aerosols, or atmospheric turbulence.

## Methods

### Monte-Carlo implementation of polarimetric mie scattering

A light beam propagating through a turbid media is subject to attenuation and scattering such that transmission is given by $t=\exp (-({\mu }_{a}+{\mu }_{s})z)$, where *μ*_*a*_ and *μ*_*s*_ are the absorption and scattering extinction coefficients respectively and *z* is the distance travelled in the medium. Absorption can be modelled simply as a reduction of photon weight (or ray intensity), reducing the total energy of the system. During scattering, energy is conserved and only the direction of ray propagation is changed, producing a far-field intensity angular distribution determined by the scattering properties of the media. To approximate Radiative Transfer in multiple scattering scenarios, a Monte-Carlo (MC) approach follows ray optics, and assumes scattering events are mutually independent. Each scattering event consists of elastic scattering defined in three steps: (a) location of the event, (b) change of direction of propagation by scattering, (c) change of electromagnetic field (determining polarisation and phase); and possible absorption.

The probability that a photon (or ray) scatters after travelling a distance *z* follows an exponential probability distribution function defined by a mean-free path, ${\mu }_{s}^{-1}$; and this statistically resembles beam extinction due to scattering after sufficient photons are included. When a photon is scattered, the new propagation direction is calculated with reference to the far-field intensity distribution that a single scatterer would produce from an incoming light beam: it has a general dependence on both the scattering and azimuthal angles. The normalised far-field intensity is called the *phase function* and, within a MC framework, it is assigned to be the probability distribution function of the scattering direction, such that after launching sufficient photons the far-field intensity is reproduced.

**Figure 4:**
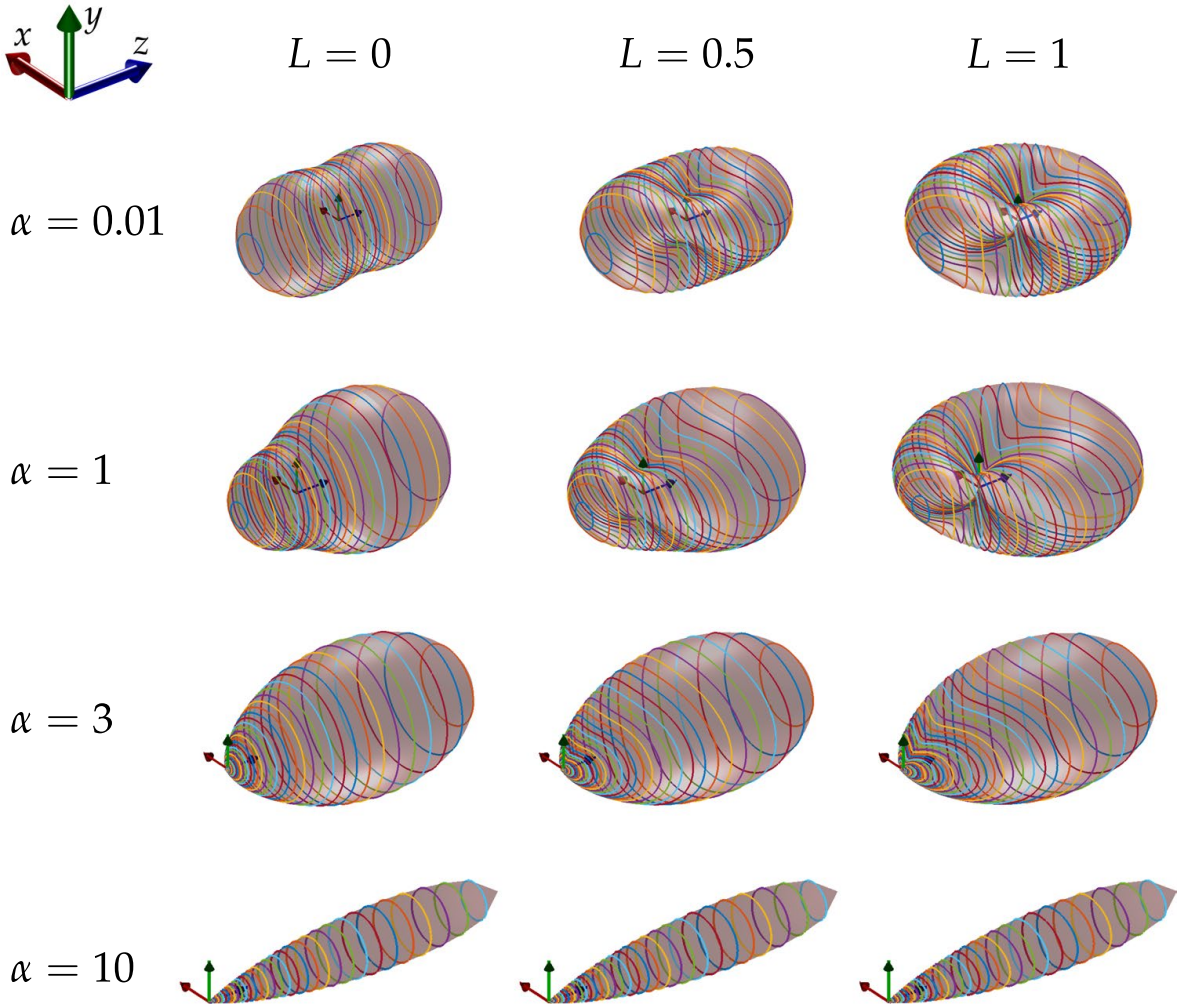
Geometry of a scattering event. Left diagram: incident ray vector ${\hat{{\bf{e}}}}_{p}^{i}$, and scattered ray vector ${\hat{{\bf{e}}}}_{p}^{s}$, define the scattering plane common to both vectors and *θ* is the scattering angle. Planes for defining polarisation states are represented by the red and blue squares. Centre diagram: the orthonormal co-ordinate systems for ${\hat{{\bf{e}}}}_{p}^{i}$ and ${\hat{{\bf{e}}}}_{p}^{s}$; the *r* direction, common to both systems, is orthogonal to the scattering plane; the propagation direction *p* and the orthogonal $\ell$ direction for the scattered photon are rotated by *θ* in the scattering plane with respect to the incident ray. Right diagram: the electric field **E**^*i*^ of the incident ray (blue) and **E**^*s*^ for the scattered ray (red) are decomposed into the respective *r* and $\ell$ directions, with respect to the azimuthal angle *ϕ*; as for the left diagram, the polarisation planes are shaded red and blue.

The nature of a scattering event is represented in Fig. 4. In the figure, the incident ray propagates in the direction of the unit vector ${\hat{{\bf{e}}}}_{p}^{i}$ represented by the blue arrow in the left-most diagram, and is scattered at the location represented by the black dot towards the new scattered direction ${\hat{{\bf{e}}}}_{p}^{s}$ represented by the red arrow. These vectors determine the scattering plane (drawn in green in the right-most diagram), defined by the scattering angle *θ*. The plane of polarisation of each ray (represented by the blue and red planes, where the electric field resides, perpendicular to the propagation directions) is defined by two unit vectors in the directions parallel and perpendicular to the scattering plane, and therefore $({\hat{{\bf{e}}}}_{p}^{i},{\hat{{\bf{e}}}}_{r}^{i},{\hat{{\bf{e}}}}_{\ell }^{i})$ and $({\hat{{\bf{e}}}}_{p}^{s},{\hat{{\bf{e}}}}_{r}^{s},{\hat{{\bf{e}}}}_{\ell }^{s})$ are the orthonormal vector basis oriented with the incident and scattered rays respectively, where the propagation, perpendicular and parallel directions are denoted by subindices *p*, *r* and $\ell$ respectively. The electric field of each ray (confined in the polarisation plane) can be decomposed into the parallel and perpendicular components, ${{\bf{E}}}^{i}={E}_{\ell }^{i}{\hat{{\bf{e}}}}_{\ell }^{i}+{E}_{r}^{i}{\hat{{\bf{e}}}}_{r}^{i}$ and ${{\bf{E}}}^{s}={E}_{\ell }^{s}{\hat{{\bf{e}}}}_{\ell }^{s}+{E}_{r}^{s}{\hat{{\bf{e}}}}_{r}^{s}$, where the mutual phases of the complex electric fields completely define arbitrary states of polarisation.

When a ray scatters towards the new direction of propagation, its electric field can be calculated as,

1 $$(\begin{array}{c}{E}_{\ell }^{s}\\ {E}_{r}^{s}\end{array})=N(\begin{array}{cc}{S}_{2}(\theta ,\varphi ) & {S}_{3}(\theta ,\varphi )\\ {S}_{4}(\theta ,\varphi ) & {S}_{1}(\theta ,\varphi )\end{array})(\begin{array}{c}{E}_{\ell }^{i}\\ {E}_{r}^{i}\end{array})$$

where ${({E}_{\ell }^{i},{E}_{r}^{i})}^{{\rm T}}$ and ${({E}_{\ell }^{s},{E}_{r}^{s})}^{{\rm T}}$ are the complex amplitudes of the orthogonal parallel and perpendicular components of the electric field of the incident and scattered rays respectively (see Fig. 4), elements *S*_1_, *S*_2_, *S*_3_ and *S*_4_ form the amplitude scattering matrix, and *N* is a normalisation constant that preserves the intensity of the ray.

Note that the elements of the amplitude scattering matrix are in general complex numbers affecting the phase of the electric field components, and hence the state of polarisation of the scattered ray in general changes. Symmetry properties simplify this matrix in various ways^43^. In particular, for spherical particles *S*_3_ = *S*_4_ = 0, and *S*_1_ and *S*_2_ do not have azimuthal dependence. This is the case we consider here. Equation (1) then reduces to,

2 $$\{\begin{array}{rcl}{E}_{\ell }^{s} & = & N\,{S}_{2}(\theta )\,{E}_{\ell }^{i}\\ {E}_{r}^{s} & = & N\,{S}_{1}(\theta )\,{E}_{r}^{i}\end{array}$$

Rigorous solution of Maxwell’s equations to provide *S*_1_(*θ*) and *S*_2_(*θ*) for spherical particles is the well-known Mie theory^43,44^. That is, given the refractive indices of the medium and scattering particle, the wavelength of light, and particle size, *S*_1_ and *S*_2_ can be calculated analytically (despite some complexity). Furthermore, Mie theory provides the scattering (and absorption if complex index of refraction of the particle is used) efficiency of the scatterers, which determine the extinction of the medium given the concentration and geometry of the particles. Many implementations of Mie calculations are available, in particular we highlight that of Wiscombe^45,46^ as has been well tested within the community (Mie calculations involve computation of infinite summation series, and accuracy and efficiency are important issues).

Importantly, even though the amplitude scattering matrix does not have azimuthal dependence, the phase function does if the incident ray has non-zero degree of linear polarisation. Its form can be written as^11^,

3 $$F(\theta ,\varphi )=A[{S}_{2}^{2}+{S}_{1}^{2}+L({S}_{2}^{2}-{S}_{1}^{2})\cos \,\mathrm{(2}\varphi )]$$

where $$L=\sqrt{{Q}^{2}+{U}^{2}}/I$$ is the degree of linear polarisation of the incident ray, $$I={E}_{\ell }{E}_{\ell }^{\ast }+{E}_{r}{E}_{r}^{\ast }$$, $$Q={E}_{\ell }{E}_{\ell }^{\ast }-$$$${E}_{r}{E}_{r}^{\ast }$$ and $$U={E}_{\ell }{E}_{r}^{\ast }+{E}_{r}{E}_{\ell }^{\ast }$$ (^*^ denoting complex conjugation) are the first three Stokes parameters, *ϕ* is the azimuthal angle defined with reference to the long axis of the incident polarisation ellipse, and *A* is a normalisation constant such that,

4 $$\int \,F(\theta ,\varphi )\,\sin \,\theta \,d\theta \,d\varphi =1$$

The phase function for Mie scattering is bivariate (does not have azimuthal symmetry for *L* ≠ 0), is polarisation dependent, and cannot be analytically inverted. The issue of sampling a (*θ*, *ϕ*) doublet with the appropriate probability is therefore a key step. Previous approaches employ some form of rejection sampling^47^ and/or ignore the polarisation dependence to allow pre-computation of the phase function^23,29^, but can be computational inefficient or accrue important inaccuracies. We employ a customised dynamic look up table algorithm, that provides efficient random sampling according to Eq. (3), which is described in Section “Angle sampling from Mie phase function”.

**Figure 5:**
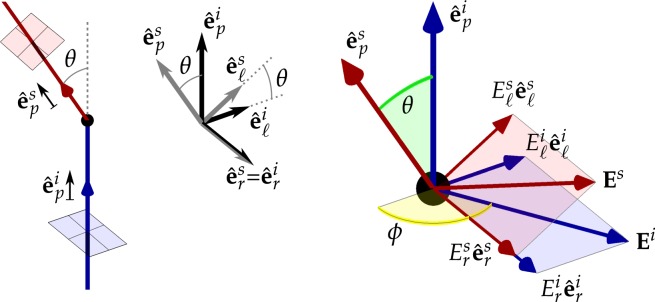
Scattering diagrams. Plots of scattering diagrams depicting the phase functions for Mie scattering, for various particle size parameters, *α* = *πdn*/*λ* (*d* is the particle size, *n* the index of refraction of the medium, and *λ* the wavelength of the light) and degrees of linear polarisation. The incident ray propagates along *z* axis and scatters at the origin of the axes. The polarised component of the partially polarised incident light is oriented with the major axis in the *y* direction. The surfaces correspond to the phase function and coloured lines are contours of constant *θ*.

Plots of Eq. (3), known as scattering diagrams, are shown in Fig. 5 for different particle sizes (which yield different anisotropy values) and degrees of linear polarisation. For the special case of very small particle sizes, the Rayleigh scattering regime is reproduced^48^, which simplifies the amplitude scattering coefficients to *S*_1_ = 1 and *S*_2_ = cos*θ* and the phase function can be written as,

5 $$F(\theta ,\varphi )=\mathrm{3(4}\pi {)}^{-1}({\cos }^{2}\theta +1-L\,{\sin }^{2}\theta \,\cos \,2\varphi )$$

which is in agreement with the first row in Fig. 5. For non-linearly-polarised light (*L* = 0), this further reduces to,

6 $$F(\theta )=3{(4\pi )}^{-1}({\cos }^{2}\theta +1)$$

which is the commonly known form of the Rayleigh phase function^43^.

### Angle sampling from Mie phase function

Since the phase function for Mie Scattering, Eq. (3), cannot be analytically inverted (*S*_1_ and *S*_2_ depend on *θ* through series expansions, and there is a dependency on an *a priori* unknown *L*), the issue of sampling a (*θ*, *ϕ*) doublet with the appropriate probability is a key step for accurate MC. The heuristic Henyey-Greenstein phase function^49^ has been widely used because is simple and is conveniently invertible to sample the new direction of polarisation of a scattered ray. Importantly, however, the Henyey-Greenstein phase function is not capable of tracking the polarisation of the scattered rays and therefore can yield highly inaccurate solutions when polarisation is important; namely when the number of scattering events is small, such as can be the case for imaging with a confocal microscope, laser scanning ophthalmoscope, optical coherence tomography, polarimetric imaging in general and modelling of speckle phenomena. The characteristic quadrifolium intensity patterns that are observed in practice from backscattering (see characteristic Mueller matrices in Supplementary Fig. 7) and underpin observed speckle patterns, are polarisation-dependent and cannot be modelled using a Henyey-Greenstein phase function.

We propose and employ an approach based on the separation of the phase function in Eq. (3) into two terms, *F*_1_ and *F*_2_,

7 $${F}_{1}={S}_{2}^{2}+{S}_{1}^{2}$$

8 $${F}_{2}=({S}_{2}^{2}-{S}_{1}^{2})\cos \,\mathrm{(2}\varphi )$$

such that

9 $$F(\theta ,\varphi )\propto {F}_{1}+L\cdot {F}_{2}$$

We then sample *θ* and *ϕ* yielding two ordered sets of the scattering and azimuthal angles,

10 $$\begin{array}{cc}{\theta }_{i}=\arccos (\frac{\mathrm{2(}i-\mathrm{1)}}{m-1}-1) & {\rm{for}}\,i=\mathrm{1,}\,\ldots ,\,m\end{array}$$

11 $$\begin{array}{cc}{\varphi }_{j}=\frac{2\pi (j-\mathrm{1)}}{n} & {\rm{for}}\,j=\mathrm{1,}\,\ldots ,\,n\end{array}$$

12 $$\begin{array}{c}\begin{array}{cc}{\omega }_{k}=({\theta }_{i},{\varphi }_{j}) & {\rm{for}}\,k=i+m(j-\mathrm{1)}\end{array}\\ =\,\mathrm{1,}\,\ldots ,\,mn\end{array}$$

where cos (*θ*) and *ϕ* are uniformly sampled within [−1, 1] and [0, 2*π*) ranges with *m* and *n* samples respectively, and the set of doublets {*ω*_*k*_} = {*ϕ*_*j*_} × {*θ*_*i*_} is the Cartesian product of the two sets and comprises *mn* samples in lexicographical order.

We denote by *F*_1_[*k*] and *F*_2_[*k*] the values of the functions sampled at *ω*_*k*_, and compute their numerical integrals *G*_1_[*k*] and *G*_2_[*k*] using the trapezoidal rule. Note that these integrals correspond to the unnormalised cumulative probability of the joint distribution, and can be pre-computed.

To sample (*θ*, *ϕ*) at each scattering event given *L* (which is readily calculated from the incident ray) we calculate a boundary number *r*_max_ = *G*_1_[*mn*] + *L* · *G*_2_[*mn*], generate a random number *r* in the range 0 ≤ *r* ≤ *r*_max_ and find *k* such that *G*_1_[*k*] + *LG*_2_[*k*] ≤ *r* < *G*_1_[*k* + 1] + *LG*_2_[*k* + 1], using binary search. Actual values of (*θ*, *ϕ*) are finally interpolated by calculating *θ* = *θ*_*i*_ + *r*_*θ*_(*θ*_*i*+1_ − *θ*_*i*_) and *ϕ* = *ϕ*_*j*_ + *rϕ*(*ϕ*_*j*+1_ − *ϕ*_*j*_), where (*θ*_*i*_, *ϕ*_*j*_) is the doublet corresponding to *k* and *r*_*θ*_ and *r*_*ϕ*_ are random numbers in the range [0,1]. The implementation included in the provided software employs the Mersenne-Twister algorithm^50^ to generate random numbers as it provides satisfactory accuracy and periodicity.

Three alternative commonly-used approaches to sample (*θ*, *ϕ*) doublets from the phase function are (*i*) using rejection sampling^47^, (*ii*) uniformly sampling (*θ*, *ϕ*) and *a posteriori* adjusting the photon weight to match the phase function or (*iii*) ignoring the polarisation dependence of the phase function followed by calculation and inversion using a look up table. However, the first two options are computationally inefficient (especially for longer particle sizes that yield high anisotropy scattering) whilst the latter can be inaccurate when polarisation is important, as discussed above.

The approach described here can be regarded as a dynamic look-up table, as it effectively builds a look-up table from two pre-calculated tables and the value *L*. This makes the MC simulation efficient since at each scattering event only the binary search and a single Mie calculation are required (the Mie calculation is used to find actual values of *S*_1_ and *S*_2_ for the interpolated (*θ*, *ϕ*) doublet, but can be avoided if the pre-computed values from the table are used instead), and yet the polarisation-dependent bivariate phase function is accurately reproduced without requiring rejection sampling.

### Simulation of fluorescence

We propose and employ two different approaches to simulate fluorescence. In this section we describe the first method, which is based on a probabilistic occurrence of wavelength shift from excitation to emission wavelengths for each ray that propagates within the fluorescent media. In the next section we describe the second method, which enables simulation of fluorescence excited from diffracted beams.

In the probabilistic method described in this section, a probability is assigned to each scattering event corresponding to fluorescence shift in wavelength from excitation to emission. For the fluorescent media we define a scattering mean-free path, a fluorescence mean-free path and a probability of fluorescence. When a ray enters the medium, a propagation distance is computed from an exponential probability distribution defined by the fluorescence mean-free path. The ray is propagated by this distance; then a new distance is calculated and the ray is propagated again preserving the direction of propagation, and so on. At each of this propagation segments, however, the ray may experience fluorescence with the given probability. Thus, the fluorescence mean-free path and the fluorescence probability determine a penetration depth (if beam extinction from fluorescence is not significant, the probability of fluorescence should be set low such that the penetration depth is larger than the sample dimensions). If fluorescence occurs, the ray undergoes a wavelength shift, a change in direction of propagation, and polarisation. The direction of propagation is selected isotropically, the polarisation is set to be linear with a random orientation and the phase is randomised. These properties simulate incoherent fluorescence emission.

Additionally, the ray may experience elastic scattering defined by the scattering mean-free path. To achieve this, a scattering probability is calculated, defined by the ratio of fluorescence to scattering mean-free paths. If the ray is scattered, then the new direction of propagation is calculated based on the medium properties and the phase function for Mie scattering as described in Section “Angle sampling from Mie phase function”, and polarisation is updated according to Mie theory as described in Section “Monte-Carlo implementation of polarimetric Mie scattering”. If the ray is not scattered (nor induced fluorescence), the direction of propagation is unchanged and the ray continues propagating. Through this framework, scattering and fluorescence are simulated simultaneously, and can be used to model fluorescent samples as well as simulation of imaging through a scattering medium that expresses auto-fluorescence.

Simulation of broadband fluorescence is possible by defining a set of probabilities and wavelengths that sample the required spectrum.

We provide a DLL that includes these functionalities and can be used in *Zemax-OpticStudio* through a graphical interface. This bulk-scattering DLL, installation instructions, and user manual are included in Supplementary Software 1.

### Fluorescence from diffracted beams

We propose and employ here a second method for simulation of fluorescence. It employs two subsequent Monte-Carlo runs: one to calculate the illumination or excitation 3D intensity pattern, and a second run to generate and propagate fluorescence rays. The method enables calculation of 3D diffraction patterns and use them as the excitation light. Two additional DLLs and software to implement this approach are included in Supplementary Software 2.

The first DLL is implemented as a surface within *Zemax-OpticStudio* and calculates the Angular Spectrum representation of an incoming light beam over a specified region and saves the measurement to disk. In this DLL an area on a plane is defined, and upon performing the optical ray trace, rays that hit the area are used to calculate the Angular Spectrum of the beam sampled by such rays. For this, a plane wave is assigned to each ray with the amplitude and phase accrued through the ray tracing from the light source (thus accounting for all optics, possible aberrations, apertures and other elements included in the system); and coherent interference of all recorded plane waves is used to compute the electric field at the plane.

We then use the beam propagation method to calculate the electric field (and therefore the illumination intensity pattern) in a voxelised volume of interest. A CAD-defined model of the sample to be imaged is then loaded (for example as an STL-formatted 3D meshed volume) and used to populate a table with 3D spatial locations and associated weights. The entries of the table represent voxels within the volume of interest, and the weights are the relative illumination intensities of each voxel. Only voxels that fall inside the sample’s volume and that are illuminated (and optionally with a weight above a defined threshold to avoid unnecessary computation of low-intensity contributions) are included in the table before it is saved to disk.

The second Monte-Carlo run uses the second DLL to load the table and generate rays with the defined emission wavelength(s). Simulation of broadband fluorescence is achieved through the definition of several wavelengths associated with the light-source DLL within *Zemax-OpticStudio*. The light-source DLL places the origin of the rays at random locations but spatially proportional to the weights defined in the table. Therefore, by launching many rays, fluorescence generated from the initial illumination is simulated.

The angular-spectrum DLL, the light-source DLL, user manuals, installation instructions, and *Matlab* code to generate the table are all included in Supplementary Software 2.

### Simulation of scattering in diffracted beams

One of the advantages of the angular-spectrum approach to simulate fluorescence described in the previous section is that it enables the use of more sophisticated models of the medium for calculation of the light-sheet propagation. In particular, weak scattering may be simulated through irregular fluctuations of the refractive index of the medium, which have been reported to model a variety of turbid media including biological tissue^51–53^. These fractal models can be incorporated into the beam propagation method to simulate the scattering of beams in their propagation through biological tissue^54,55^. This approach is implemented in Supplementary Software 2 and enables simulation of scattering of focused beams in biological media. A further important advantage is that it readily enables inclusion of a distinct scattering, absorption and refraction due to the sample. Scattering and absorption in both the medium and within the neuron volume were included in the example shown in Supplementary Fig. 4, where it can be appreciated how the variations of the index of refraction of the medium cause the beam to scatter. An illustration of the scanning sequence including scattering and absorption with the superimposed model of the neuron can be seen in Supplementary Video 2, where the effect of the scattering in the medium and absorption in the neuron are particularly evident.

## Supplementary information

Supplementary information

Supplementary information

Supplementary information

Supplementary information

Supplementary information

Supplementary information

Supplementary information
