# Publisher Correction: Holistic Monte-Carlo optical modelling of biological imaging

**DOI:** 10.1038/s41598-020-68533-x

**Published:** 2020-07-07

**Authors:** Guillem Carles, Paul Zammit, Andrew R. Harvey

**Affiliations:** School of Physics and Astronomy, University of Glasgow, Glasgow, G12 8QQ UK

Correction to: *Scientific Reports* https://doi.org/10.1038/s41598-019-51850-1, published online 01 November 2019

This Article contains an error in the order of the Figures. Figures 4 and 5 were published as Figures 5 and 4 respectively. The correct Figures 4 and 5 appear below as Figures 1 and 2. The Figure legends are correct.

**Figure 1:**
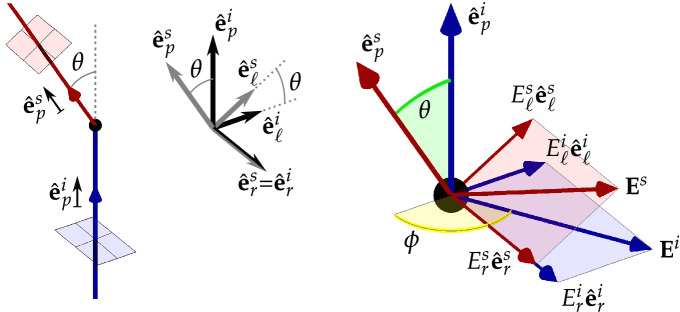
Geometry of a scattering event. Left diagram: incident ray vector ${\hat{\mathbf{e}}}_{p}^{i}$, and scattered ray vector ${\hat{\mathbf{e}}}_{p}^{s}$, define the scattering plane common to both vectors and *θ* is the scattering angle. Planes for defining polarisation states are represented by the red and blue squares. Centre diagram: the orthonormal co-ordinate systems for ${\hat{\mathbf{e}}}_{p}^{i}$ and ${\hat{\mathbf{e}}}_{p}^{s}$; the *r* direction, common to both systems, is orthogonal to the scattering plane; the propagation direction *p* and the orthogonal $\ell$ direction for the scattered photon are rotated by *θ* in the scattering plane with respect to the incident ray. Right diagram: the electric field **E**^*i*^ of the incident ray (blue) and **E**^*s*^ for the scattered ray (red) are decomposed into the respective *r* and $\ell$ directions, with respect to the azimuthal angle $\phi$; as for the left diagram, the polarisation planes are shaded red and blue.

**Figure 2:**
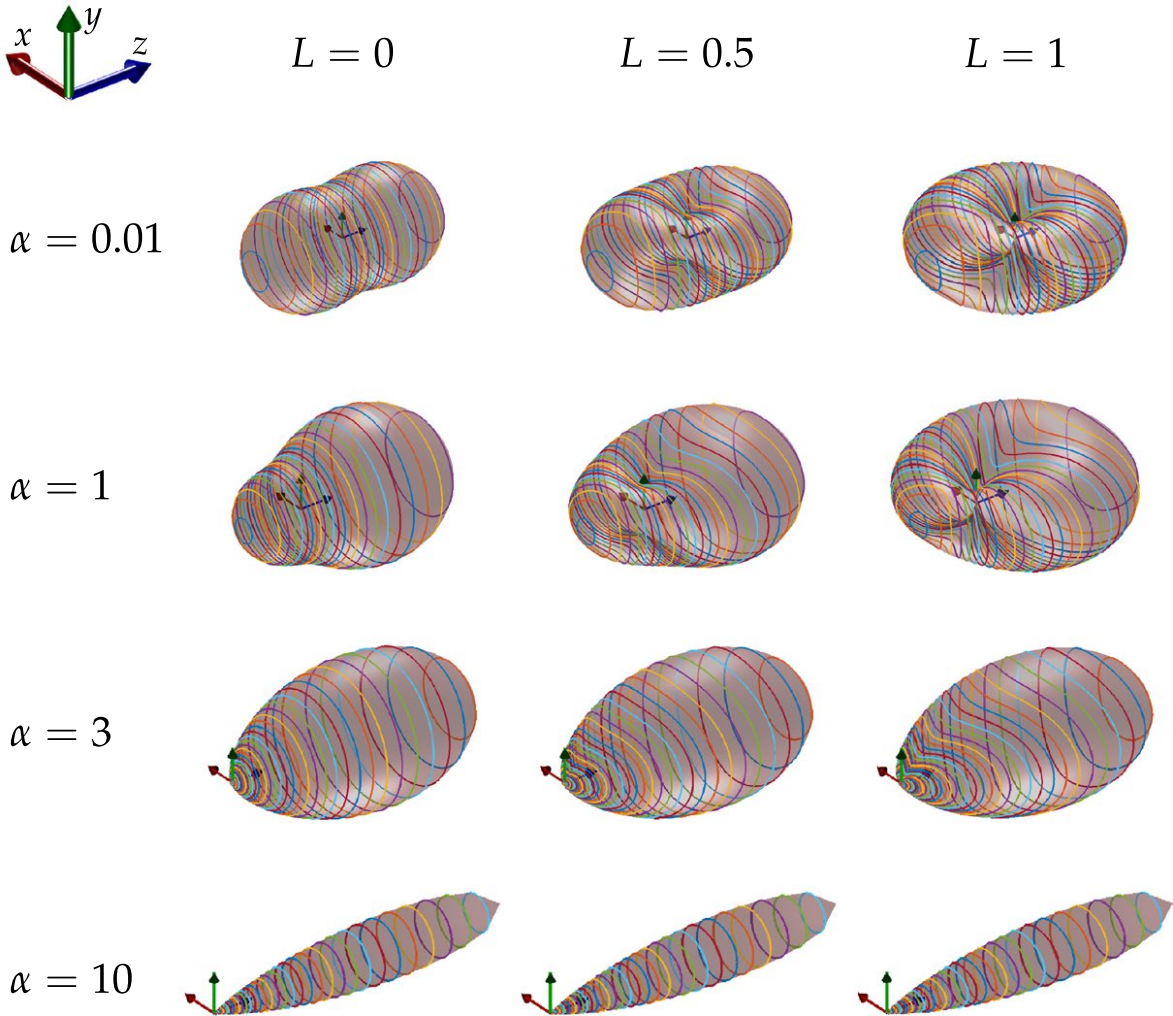
Scattering diagrams. Plots of scattering diagrams depicting the phase functions for Mie scattering, for various particle size parameters, *α* = *πdn*/*λ* (*d* is the particle size, *n* the index of refraction of the medium, and *λ* the wavelength of the light) and degrees of linear polarisation. The incident ray propagates along *z* axis and scatters at the origin of the axes. The polarised component of the partially polarised incident light is oriented with the major axis in the *y* direction. The surfaces correspond to the phase function and coloured lines are contours of constant *θ*.

