# Supplementary material for: Holistic Monte-Carlo optical modelling of biological imaging
Source: Sci Rep. 2019 Nov 1;9:15832. doi: 10.1038/s41598-019-51850-1 (PMC6825179; doi:10.1038/s41598-019-51850-1)
Supplement: Supplementary file 1 — Supplementary information [file 41598_2019_51850_MOESM1_ESM.pdf]

Supplementary information:
Holistic Monte-Carlo optical modelling of biological imaging

Guillem Carles, Paul Zammit, and Andrew R. Harvey

School of Physics and Astronomy, University of Glasgow, Glasgow G12 8QQ, UK.

1 Supplementary methods

The simulation of the micro-endoscope microscope in Fig. 1 of the main paper exemplifies a common requirement to understand and rigorously model image formation by a complex imaging system for imaging of complex, scattering and fluorescent 3D biological samples. We demonstrate holistic Monte-Carlo optical modelling (HMCOM) of imaging of a labelled neuron embedded within scattering tissue, using a miniaturised microscope employing a graded-index (GRIN) lens. The system comprises an extended light source (such as an LED) and collimating lens, dichroic mirror, GRIN-lens objective immersed within a biological scattering medium, an achromatic-doublet tube lens and pixelated detector. The dichroic mirror is a 4mm-diameter round window of N-BK7 glass with 1mm thickness, anti-reflection coated on the front side and semi-reflective coating on the reverse side so as to transmit the 488nm excitation light, and to reflect fluorescence with a central wavelength of 510nm. See Supplementary Fig. 1 for a depiction of the discrete wavelengths employed to simulate the continuous excitation, fluorescence and filter-transmission spectra. This spectral sampling enables appropriate intensity weighting of the image and incorporation of chromatic aberrations into the model.

The GRIN lens is a 3.8mm-long, 1.8mm-diameter cylinder with refractive index variation $n(r) = n_0 + n_2 r^2 + n_4 r^4$ where r is the radial coordinate and $n_0 = 1.62893$, $n_2 = -0.10673\,\mathrm{mm}^{-2}$, $n_4 = 0.00440\,\mathrm{mm}^{-4}$. The GRIN lens is immersed in water containing scattering particles of 3.47 µm diameter and refractive index of $n = 1.47$. The mean-free path between scattering events is controlled by the scattering coefficient μ_s, which is set to $0.5\,\mathrm{mm}^{-1}$, $5\,\mathrm{mm}^{-1}$ and $10\,\mathrm{mm}^{-1}$ for the examples shown in Fig. 1 of the main paper. The sample is a CAD-defined model of a neuron imported as a stereolithography (STL) formatted volume. Fluorescence of the neuron and scattering in the medium are simulated using the method described in Section "Simulation of fluorescence" of the main paper, using a probability of fluorescence of 50% per scattering event, a fluorescence mean-free path of 0.2 µm, and a scattering coefficient of $\mu_s = 10\,\mathrm{mm}^{-1}$. The achromatic-doublet tube lens ($f = 7.5\,\mathrm{mm}$, AC050-008-A, *Thorlabs Ltd*, using the optical prescription from the supplier) focuses light exiting the GRIN lens onto a pixelated detector that records all rays incident on a 512×512 pixel image, integrating the rays falling on each pixel. The simulation involved launching 1.6×10^{10} photons yielding 6.16×10^{6} fluorescent photons that reached the detector (when $\mu_s = 0.5\,\mathrm{mm}^{-1}$). Axial displacements of the detector from nominal best focus simulated depth-of-field effects and defocus-related blurring as shown in Supplementary Fig. 2. Image contrast reduction due to scattering in the medium was simulated by varying the scattering coefficient, see Supplementary Fig. 3, and plots of image intensity profiles quantify how scattering strength affects image contrast.

Fluorescence and scattering in the neuron and medium employed a rigorous model of Mie scattering implemented using a custom Dynamic Link Library (DLL) (Supplementary Software 1), which can be easily installed and used within the optical raytrace software *Zemax-OpticStudio*. Rigorous tracking of the polarisation of scattered light is not currently implemented in *Zemax-OpticStudio*, but is implemented here through this DLL. The theory and implementation are described in Section "Methods" of the main paper. Validation results of the full polarimetric description, consisting of the Mueller-matrix images calculated for backscattering of laser light focused onto a slab of turbid material are described in Section 2 and depicted in Supplementary Fig. 9. An illustrative example of the modelling of polarimetric image formation of a three-dimensional CAD structure employing scattered light is described in Section 3 and results are shown in Supplementary Fig. 10.

The simulation of the Light-Sheet Microscope in Fig. 2 of the main paper demonstrates the integration of diffraction (in this case for the fluorescence excitation) with ray tracing of the optical system and Monte-Carlo modelling for scattering and fluorescence emission within the sample. To simulate diffraction of the light-sheet illumination and the imaging of fluorescence, we employed the method described in Section "Fluorescence from diffracted beams" of the main paper. In summary, the method employs three steps: calculation of the 3D illumination pattern, calculation of the resulting 3D spatial distribution of the fluorescence, and tracing of fluorescence rays to simulate imaging. A light source was implemented by launching a collimated

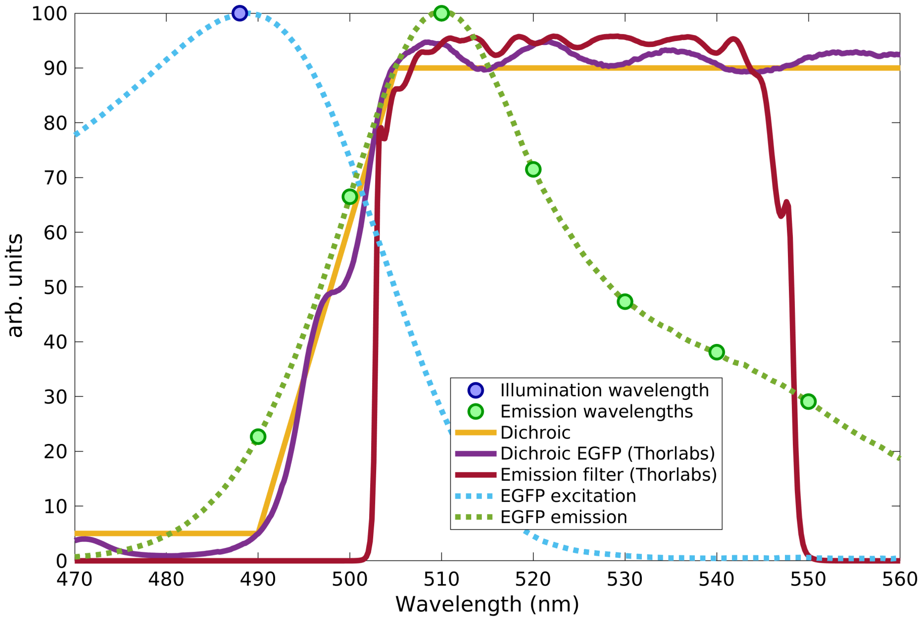

Supplementary Figure 1: Summary of spectra and simulation wavelengths. Excitation and emission spectra of EGFP; the circles show the relative weightings of discrete wavelengths employed for the simulations in Fig. 1 and Fig. 2 of the main paper. The reflection and transmission curves of the dichroic coating employed in Fig. 1 are also plotted along with representative transmission spectra for the dichroic mirror and emission filter (from *Thorlabs Ltd* standard components).

beam implemented as 10^5 rays of wavelength $\lambda = 488\,\text{nm}$ filling the 6mm-diameter aperture of the objective. Horizontal focusing by the cylindrical lens to the back focal plane of the objective, created the light sheet at the sample volume. A subset of the rays are drawn in blue in Fig. 2(a). This sampling enables calculation of the Angular Spectrum of the beam, and the diffractive beam-propagation method may then be used to calculate the electric field within the volume of interest. In this example, the electric field was computed at the xy-plane (at $|x,y| \leq 100\,\mu\text{m}$ in a $0.1\,\mu\text{m}$-spaced grid, and $z = -100\,\mu\text{m}$ where $z = 0$ is the nominal focus) and propagated $200\,\mu\text{m}$ in the positive z direction, sampled at $0.2\,\mu\text{m}$ steps). The electric field is thus computed in a 1 giga-voxel volume of $2003\,\mu\text{m}^3$. The resultant 3D intensity pattern of this process is shown in Fig. 2(b). Additionally, this approach enables simulation of medium turbidity (scattering of the diffracted beam) and sample refraction as is described in Section "Fluorescence from diffracted beams" of the main paper. Example results are shown in Supplementary Fig. 4 and Supplementary Video 1.

The second step is the calculation of the 3D fluorescence map; for this, we construct a table containing 3D locations and associated relative intensities, for only those voxels that are inside the sample volume (that is, those that will fluoresce). To reduce unnecessary computation we also include only voxels for which the excitation intensity is above a threshold (in this example set to 0.1% of the maximum illumination intensity). The third step is to launch fluorescence rays from all voxels corresponding to locations within a fluorescing structure, using the table stored in the previous step and a light-source DLL that we have implemented to yield locations for the rays origins which are proportional to the relative intensity of the illumination. The direction of propagation, polarisation and phase are randomised to simulate incoherent emission. In this example, 10^8 rays were traced with a wavelength sampling that simulates the broadband-emission spectrum of EGFP (central wavelength $\lambda = 510\,\text{nm}$, see Supplementary Fig. 1), and an example subset of rays is illustrated in green in Fig. 2(a). This example system employs a cylindrical lens with focal length of 50 mm (LJ1821L1-A, *Thorlabs Ltd*) and a compound $12.8\times$/0.25NA infinity-corrected illumination objective comprising five lens elements. The light sheet propagates through a medium with absorption coefficient $\mu_a = 4.6\,\text{mm}^{-1}$.

The imaging arm of the light-sheet microscope focuses the fluorescence-emission rays onto a detector with 1000×1000 pixels. We employed a compound $20\times$/0.4NA objective comprising nine lens elements. These three simulation steps generate one image of the sample scanning process, and so they are repeated, as the sample is scanned through the light sheet, to yield a 3D scan of the sample. In this example, 301 slices were generated for the reconstruction of the neuron cell shown in Fig. 2(c). The DLL for calculation of the angular spectrum of a beam, sample *Matlab* code to implement the beam propagation, and a DLL for generating fluorescent rays are provided in Supplementary Software 2.

We discuss next the simulation of the Scanning Laser Ophthalmoscope (SLO) in Fig. 3 of the main paper. The SLO is

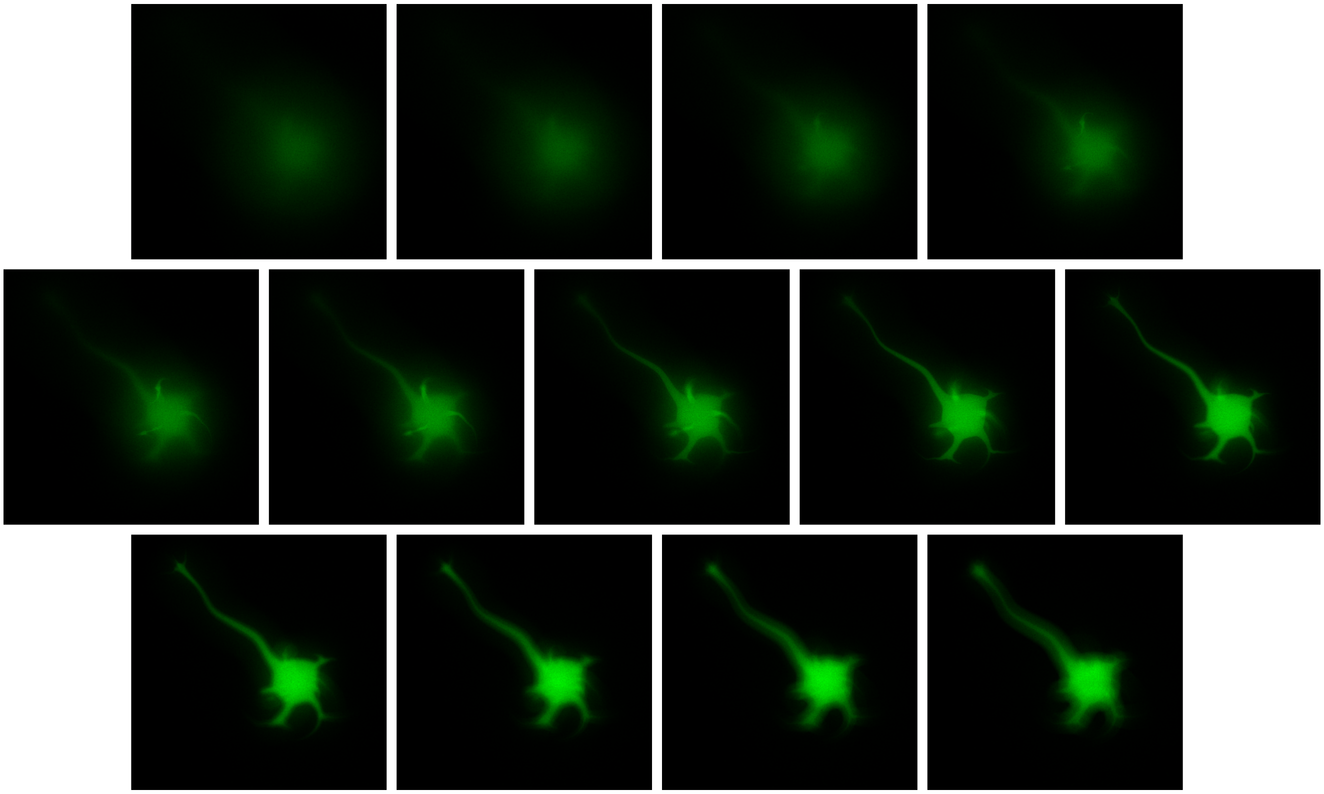

Supplementary Figure 2: Demonstration of depth of field in fluorescence imaging with a micro-endoscope microscope. Simulation results for the example of imaging a neuron cell through a micro-endoscope microscope demonstrating out-of-focus blurring due to the modest depth-of-field. Each image from left to right and top to bottom is focused at a different plane, achieved by axial movement of the detector, corresponding to focal planes at the sample ranging from $-20\,\mu m$ (top-left image) to $20\,\mu m$ (bottom-right image).

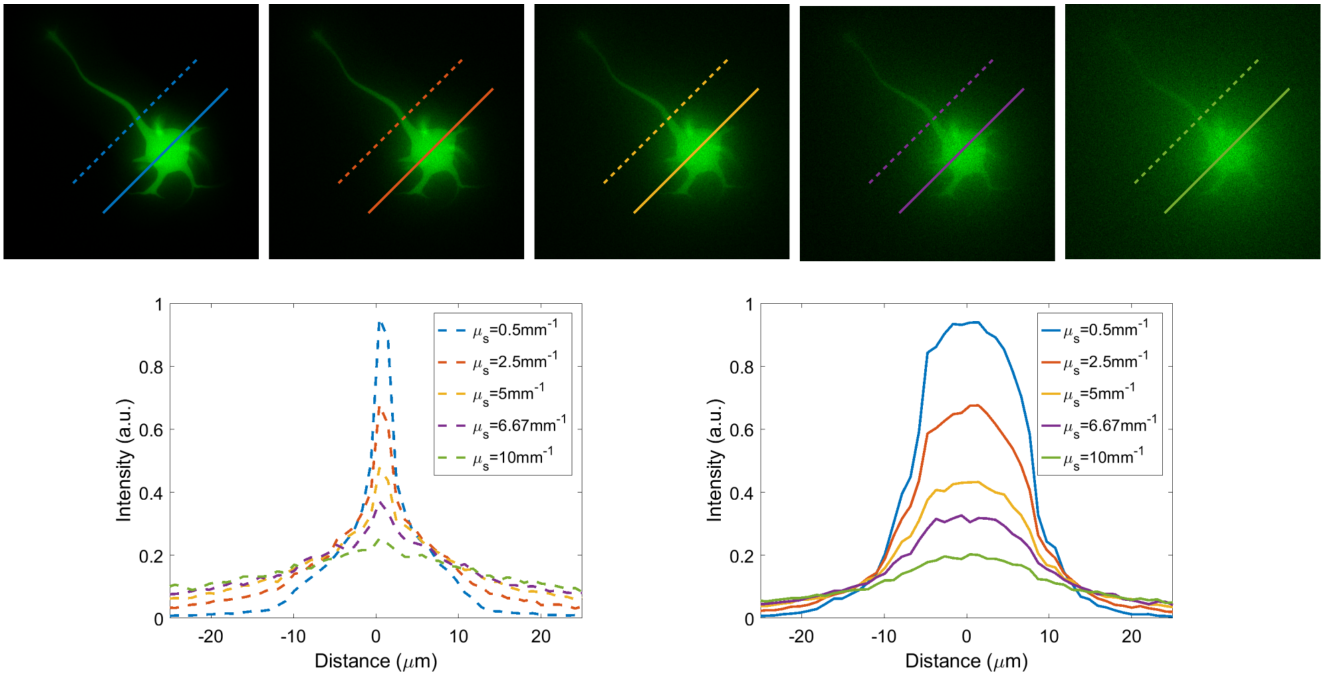

Supplementary Figure 3: Effect of scattering in the medium. Simulated images of a neuron recorded with a micro-endoscope microscope demonstrating modelling of a range of medium scattering strengths. The scattering parameter of the medium μ_s is, from left to right, 0.5 mm^{-1}, 2.5 mm^{-1}, 5 mm^{-1}, 6.67 mm^{-1}, and 10 mm^{-1}. Image intensity profiles are plotted in the lower graphs (normalised by total intensity at detector).

illustrative of a general class of imaging system with a relatively high complexity, which aims to control how light-paths within the biological sample contribute to image formation. When used in confocal mode the SLO operates in a similar way to a confocal microscope to reject scattered light, hence increasing contrast and reducing depth of field. HMCOM enables simulation of image formation involving complex light-tissue interaction and accounting for optical effects of both the illumination and imaging optics of the instrument. For purposes of generality we simulate a generic SLO, including a two-wavelength light source that launches collimated Gaussian-beam laser illumination with full widths of 700 µm (1/e^2 intensity) directed into the eye via a small axial coupling mirror as shown in Fig. 3(a).

It is common for SLOs to employ multiple laser wavelengths to enable rendering of an image with a natural coloured appearance: we have employed two wavelengths of 532 nm and 633 nm corresponding to commonly available green and red laser wavelengths that provide a useful colour-like image. The eye was implemented using a schematic-eye model comprised of the anterior segment (cornea, anterior chamber, iris and lens), vitreous humour, retinal layers and sclera. The schematic eye focuses the illumination to a Gaussian spot of width 20 µm at the retina. The eye is diffraction limited for pupil diameters less than 2 mm[1] and so without loss of accuracy and for computational efficiency our implementation locates the Gaussian-beam light source directly in front at the retina. The use of an aberrated illumination spot (for example, due to a wider illumination beam, as might be used with adaptive optics) is also possible, but is not necessary in this generic model.

The scattering retinal structure at the back of the eye includes the retina (200 µm thick), the absorbent retinal pigment epithelium (10 µm thick), the blood-rich, highly-oxygenated choroid (250 µm thick) and highly-scattering, high-albedo sclera (700 µm thick). CAD-defined 3D models of networks of venules and arterioles were embedded into the retinal tissue at 70 µm (two arterial and one venule) and 130 µm (one arterial and three venule) anterior to the retinal pigment epithelium. See Section 4 for further details. For each component within the model, the index of refraction, absorption coefficient, scattering coefficient, and scattering anisotropy were defined. Following scattering and absorption within the retinal structure, a small fraction of backscattered rays exit through the pupil into the SLO, where they are focused by an aspheric achromatic lens of 50 mm focal length (item 49-665, *Edmund Optics Inc*) onto a 1mm$\times$1mm detector plane which we sample with 101$\times$101 pixels. Pixelation of the detector enables flexible post processing of the detected rays for simulation of a range of pinhole sizes; ranging from small and confocal to bucket detection. A small pinhole rejects scattered light from an extended volume around the illumination image and increases image contrast since this confocal light tends to be transmitted twice through the vasculature increasing absorption. The *quid pro quo* for the rejection of scattered light is a reduction in detected light. Conversely, non-confocal light transmitted through a large pinhole tends to have been transmitted just once through the vessel followed by extended diffusion

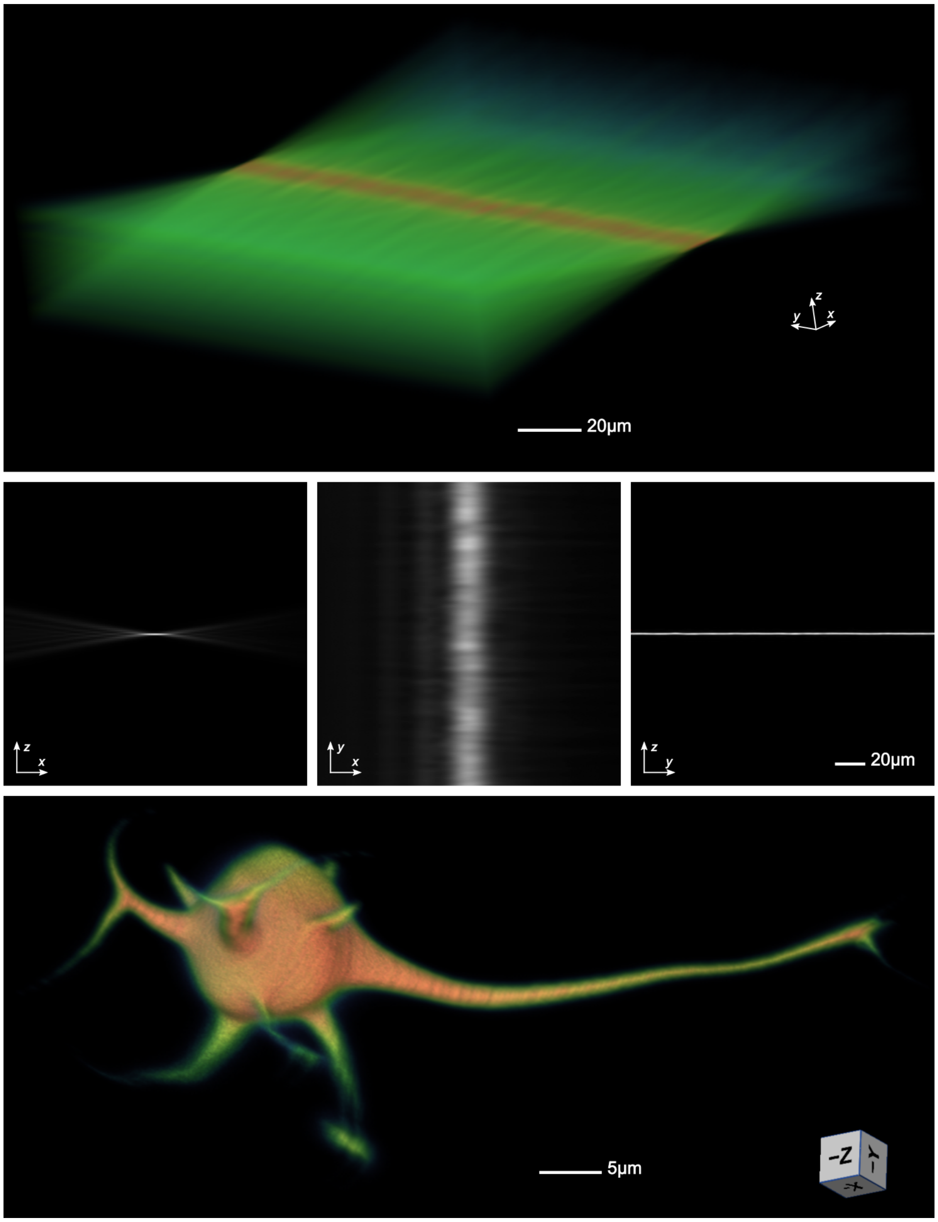

Supplementary Figure 4: Modelling of scattering in light-sheet microscopy. The diffracted illumination beam was propagated through a medium with irregular variations in refractive index to simulate scattering. The middle density plots show light intensities in the (x,z), (x,y) and (y,z) planes. The scanning sequence with the light-sheet, involving scattering and absorption by the sample, is presented in Supplementary Video 2. Scattering in the illumination creates imperfect illumination (note the irregular pattern appreciated in the (x,y) view of the light-sheet in the middle row) yielding the moderate artifacts apparent in the 3D reconstruction of the neuron shown at the bottom.

from the illumination point into the tissue neighbouring a blood vessel that is imaged onto the pinhole.

The variation of image contrast and signal-to-noise ratio with the diameter of the pinhole is shown in Supplementary Fig. 5 and Supplementary Fig. 6. SLOs employ a two-dimensional scanning mechanism to scan the illumination spot over an extended retinal area. For convenience, we instead employ the equivalent process of rotating the eye fundus about the centre of the eye ball. Aberrations in the illumination, either ocular or from the instrument, which increase with field angle, are then not included in this implementation, however these are normally not significant compared to the much larger aberrations introduced by imaging through the much higher numerical aperture of the 6 mm diameter eye pupil (that is, NA≈0.023 for the illumination compared to NA≈0.20 for imaging). A total of 10^5 rays were traced for each scanning position (generating one pixel of the output image). The scan was performed over a field of view of 48° horizontally and 33.4° vertically in steps of 0.1°. The smaller region of interest in Fig. 3(d,e) spans 12° horizontally and 9.3° vertically, and was simulated with a spacing of 0.05° in both dimensions and tracing a total of 4×10^5 rays per pixel. The optical absorption coefficient for oxygenated and deoxygenated haemoglobin differs significantly at the two illumination wavelengths. At 532 nm the absorption coefficient for haemoglobin is similar for both the highly oxygenated arterial blood and the partially oxygenated venous blood and is sufficiently high to yield high-contrast for both arteries and veins: calculation of single-pass transmissions based on the simple Beer-Lambert law are 90.5% for a typical 100 μm calibre artery (97% oxygenation) and 89.9% for a 100 μm calibre vein (70% oxygenation); at 633 nm there is a strong oximetric contrast so that the transmission and the equivalent transmissions are 2.8% for arteries and 9.3% for veins. This oxygenation sensitivity of absorption yields dissimilar contrast for arteries and veins, as can be appreciated from Fig. 3 and underpins blood oximetry. The Beer-Lambert law neglects scattering by blood cells within the vessels and furthermore scattering within the retinal structure strongly impacts on the vascular contrast that underpins retinal oximetry[2,3]. Our model enables, for the first time, a convenient and precise calculation of vascular contrast in relation to the complex light transmission processes in the retinal tissue. This paves the way to improved oximetry calculations, perhaps using inverse-Monte-Carlo methods, in which all pertinent parameters may be varied, such as vascular geometry, blood oxygenation and hematocrit, laminar-flow orientation of blood cells[4], natural variations in retinal structure and pigmentation[2,3]. As an example, of the effect of just oxygenation, we calculate retinal images for both normoxia and for mild hypoxia (10% reduction in arterial and venous blood oxygenation) as shown in Supplementary Fig. 11 and discussed in Section 5.

Our modelling above is generically applicable to modelling of retinal images close to the macula where aberrations of the eye and SLO on illumination are negligible compared to diffraction and the blurring effects of light propagation in tissue. More accurate modelling, pertinent to a specific SLO, and using proprietary optical design information, may be readily implemented, following the procedures described above for modelling fluorescence microscopes. This becomes increasingly important for wide field angles[5], for which ocular and instrumental aberrations, reflections from ocular media, vignetting and polarisation effects become more important.

To provide a comparison between our simulation and experimental results, we observe the effect of confocality on images obtained with a SLO. Confocal and non-confocal images were obtained using a modified SLO[5], and are shown in Supplementary Figures 7 and 8. The effect of confocality (mainly the contrast increase at the expense of a reduction in signal, specially at longer wavelengths) is observed as predicted by our simulations.

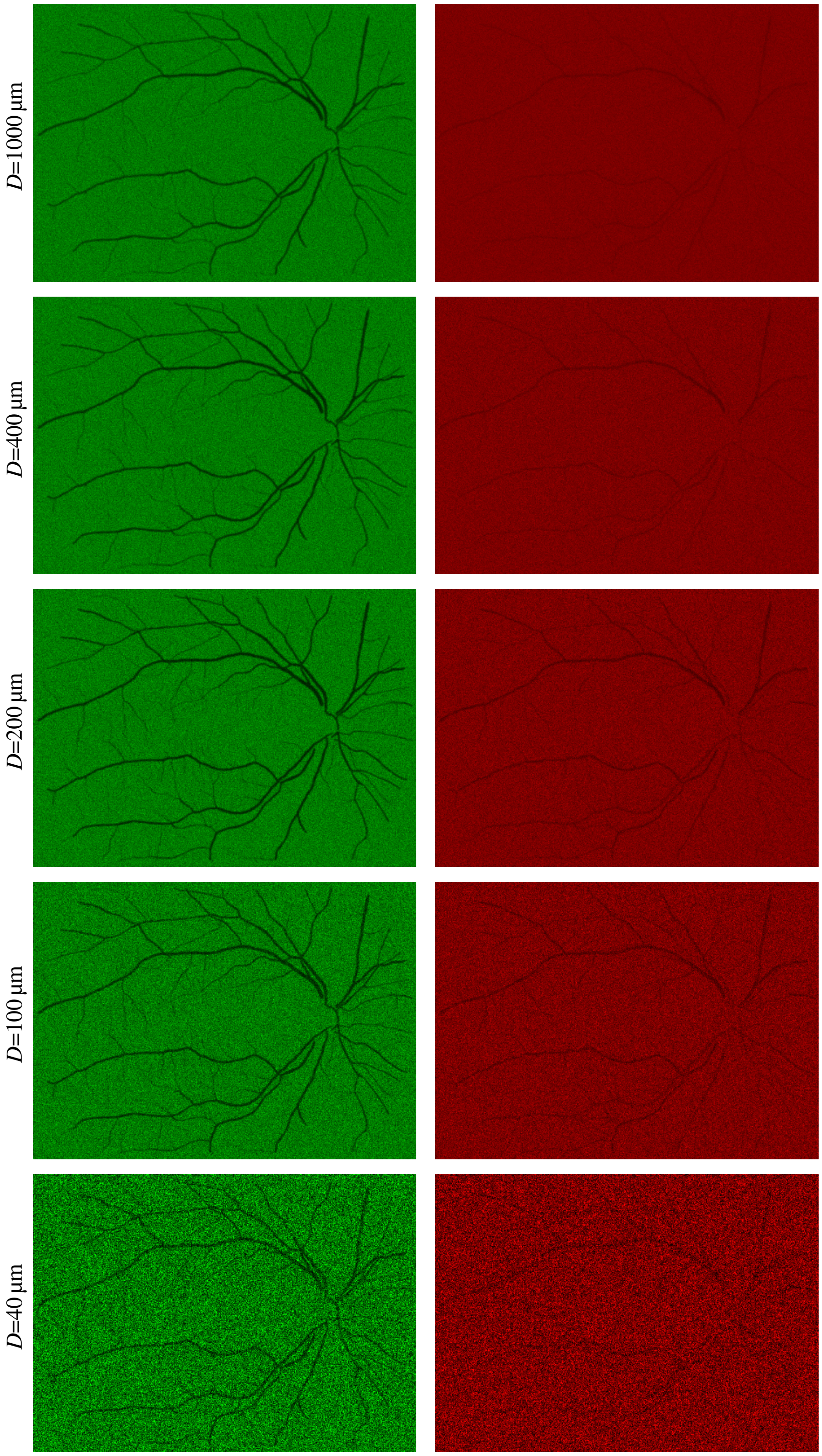

Supplementary Figure 5: Effect of the pinhole size in SLO images. Rendered images using laser wavelength of λ=532 nm (left) and λ=633 nm (right) for a range of pinhole diameters, D. Larger pinhole sizes enable more light to be collected yielding a higher signal-to-noise ratio (no noise was added to the images but the effect of the pinhole size on the Monte-Carlo simulation has a very similar effect), whereas smaller pinhole sizes (approaching a confocal configuration) provide increased image contrast due to a greater restriction on light paths (the double-pass, through-vessel transmission components are preferentially detected over single-pass light paths). This is particularly evident in the arteries at wavelength λ = 633 nm due to the lower extinction coefficient of oxygenated blood: some of the smaller-calibre arteries have very low contrast in non-confocal configurations but contrast and visibility increases with confocality since rays have a greater probability of having been transmitted twice through the artery. This is shown more clearly in Supplementary Fig. 6, which shows a small region of interest with increased sampling density.

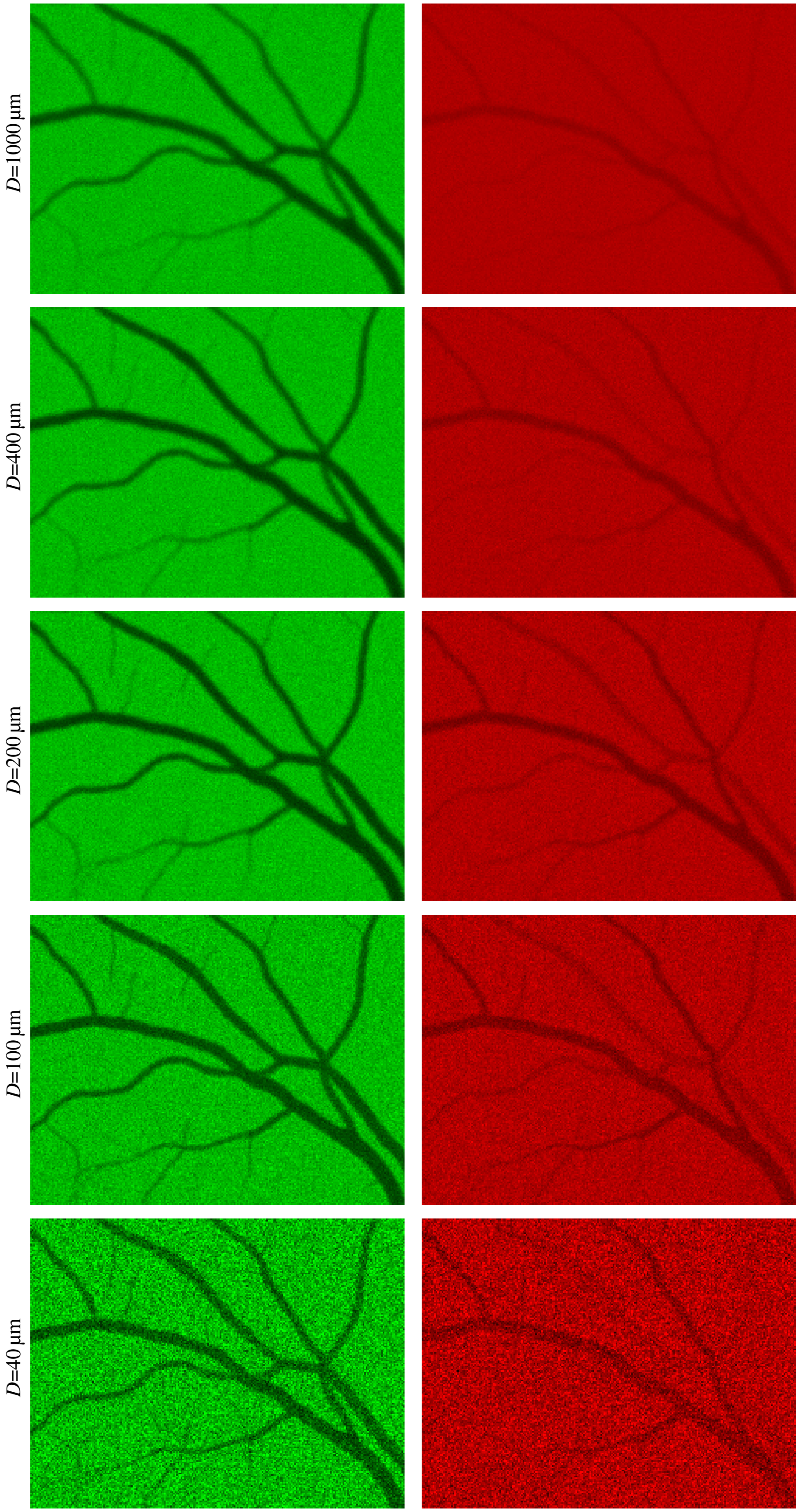

Supplementary Figure 6: Effect of the pinhole size in SLO images. Results as in Supplementary Fig. 5 for a reduced region of interest with increased sampling.

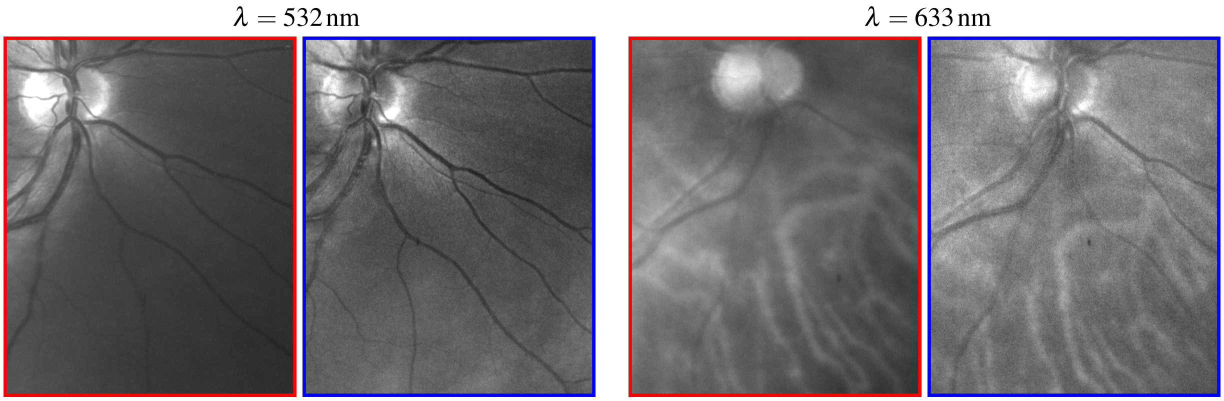

Supplementary Figure 7: Images acquired with a SLO using a non-concocal and a confocal configuration, in red and blue respectively; for illumination wavelength of 532nm (left images) and 633nm (right images). Adapted with permission from[5].

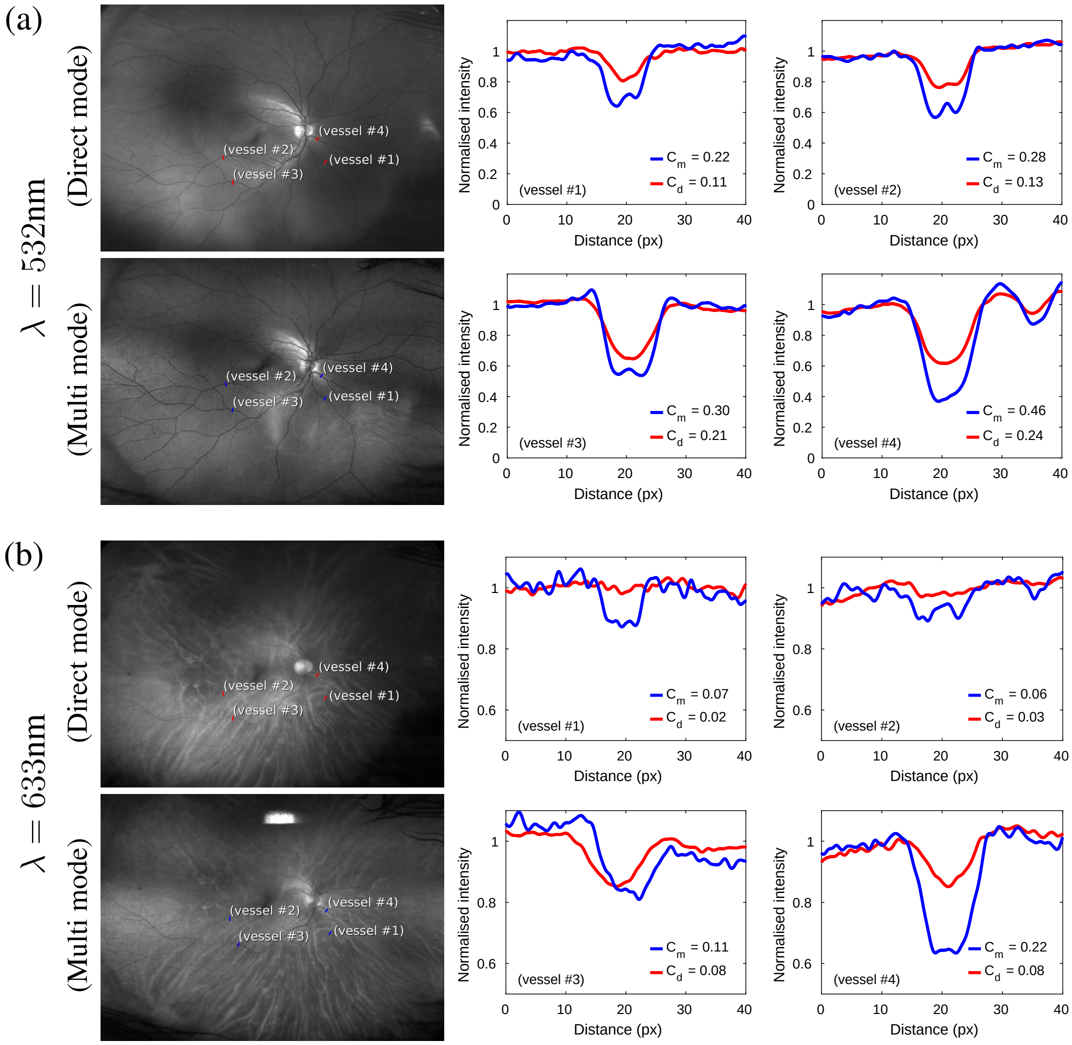

Supplementary Figure 8: Images acquired with a SLO and normalized intensity profiles across selected blood vessels, for wavelengths of (a) 532nm and (b) 633nm. Intensity profiles (and label lines in the images) in red and blue correspond to non-confocal (direct mode) and confocal (multi mode) acquisitions respectively. Calculated contrast C is shown for each profile. Reproduced with permission from[5].

2 Mueller matrices from backscattering

As a generic experiment to validate the modelling of polarisation on scattering events, we calculated the Mueller matrices that relate the input and output polarisation states of any polarisation-manipulating system: in this case a slab of turbid medium as a function of transverse location. Polarisation explains, for example, the quadrifolium pattern that forms when a linearly polarised laser beam illuminates a slab of turbid material and is observed through crossed or co-aligned polarisers[6]. The polarisation characteristic of the quadrifolium pattern arises from both geometrical effects associated with large-angle scattering and modest depolarisation due to many small-angle scattering within the scattering medium.

In our model, coherent light of wavelength λ = 520 nm illuminates a slab of water (refractive index n=1.33) with a thickness of 11.2 µm, filled with spheres (n =1.59) of diameter 0.46 µm and the mean-free path is μ_s^{-1} = 2.8 µm. Light rays scatter several times and some eventually propagate back out of the medium where are detected by the detector. Using Stokes notation, polarisation is defined by a 4-component vector, and we can relate the state of polarisation of the input (illumination) rays with the state of polarisation of the backscattered rays, through the 4×4-element Mueller matrix,

$$\mathbf{s}_b(\mathbf{r}) = \mathbf{M}(\mathbf{r})\mathbf{s}_i(\mathbf{r}_0) \tag{1}$$

where $\mathbf{s}_b(\mathbf{r})$ and $\mathbf{s}_i(\mathbf{r}_0)$ are the Stokes vectors of the backscattered and input rays respectively, $\mathbf{r}$ is the point of detection of the backscattered ray in the detector plane, $\mathbf{r}_0$ is the point of injection (in this case the centre of the detector, i.e. $\mathbf{r}_0 = \mathbf{0}$, and $\mathbf{M}(\mathbf{r})$ is the spatially-dependent Mueller matrix associated with the material.

The images shown in Supplementary Fig. 9 are the spatial maps $\mathbf{M}(\mathbf{r}) = \{\mathrm{m}_{ij}(\mathbf{r})\}$ for $i, j = 1, \ldots, 4$ representing the spatially-variant components of the Mueller matrix, and were obtained following the procedure described in[7]. The observed patterns are in accordance with previously reported simulations and measurements[7–10].

3 Polarimetric imaging example

We present here an example that illustrates a relatively complex problem that can be readily implemented, as depicted in Supplementary Fig. 10. The system consists of a CAD-defined volume of a university coat of arms that is mapped onto an artificial turbid structure containing 10 µm-diameter scattering particles with relative refractive index of 1.1 and sufficient number density to give μ_s = 100 mm^{-1}. The volume is immersed in a non-scattering medium: only the internal volume of the coat of arms is scattering. The black, blue and gold components of the coat of arms are etched to thicknesses of 35, 70 and 105 µm respectively, corresponding to about 3.5, 7 and 10.5 mean-free paths respectively. The volume is illuminated by collimated linearly-polarised light of wavelength 633 nm and a lens of focal length 50 mm forms an image of the coat of arms at a detector on a $4f$ configuration. Either crossed linear polarisers are employed for input and scattered light or an axial dot placed in the back focal plane of the lens (with no polarisers) is used to block unscattered light to yield the images shown in Supplementary Fig. 10 (b) and (c) respectively: in both cases, no light is transmitted to the detector in the absence of scattering. When only the crossed linear polarisers are used, the depolarisation of the light increases with increasing thickness and so the intensity of image features can be seen to increase; the detector images the degree of depolarisation. Note also that multiple scattering yields an extended point-spread function in the medium (known as a tissue point-spread function in biomedical imaging) resulting in blurring of the image. When only the obscuring dot without polarisers is located at the back-focal plane of the camera, only scattered light reaches the detector and increasing thicknesses of scattering medium also yields increasing brightness, however the narrower point-spread function in the medium yields less blurring. Finally, the image in (d) is formed without polarisers or axial dot, and detects unscattered light; intensity decrease is due only to scattering extinction in this case. The differences between these images highlight the importance of using the the correct polarisation model for scattering.

Three effects can be observed in this experiment as the thickness of the object increases: (*i*) there is higher (total) scattering extinction that, after undergoing polarisation changes, contributes through the lens and analyser to increase the detected intensity, (*ii*) the effective scattering anisotropy is reduced, as a higher thickness corresponds with higher degree of light diffusion, and thus increases the amount of light that is scattered at higher angles that are not captured by the lens, and (*iii*) absorption within the medium reduces intensity captured by the detector. The relative contribution of these effects will depend on the properties of the medium but, being different, a contrasted image of the object is formed and is shown in Supplementary Fig. 10(b-d).

4 Model of the eye and optical parameters in SLO simulations

The model of the eye used to simulate image acquisition in the SLO comprises the following elements: cornea, anterior chamber, iris, lens, vitreous humour, retina, arterioles, venules, retinal pigment epithelium, choroid and sclera. The optical properties of each element are summarised in Supplementary Table 1. The index of refraction of the retinal layers matches the vitreous humour.

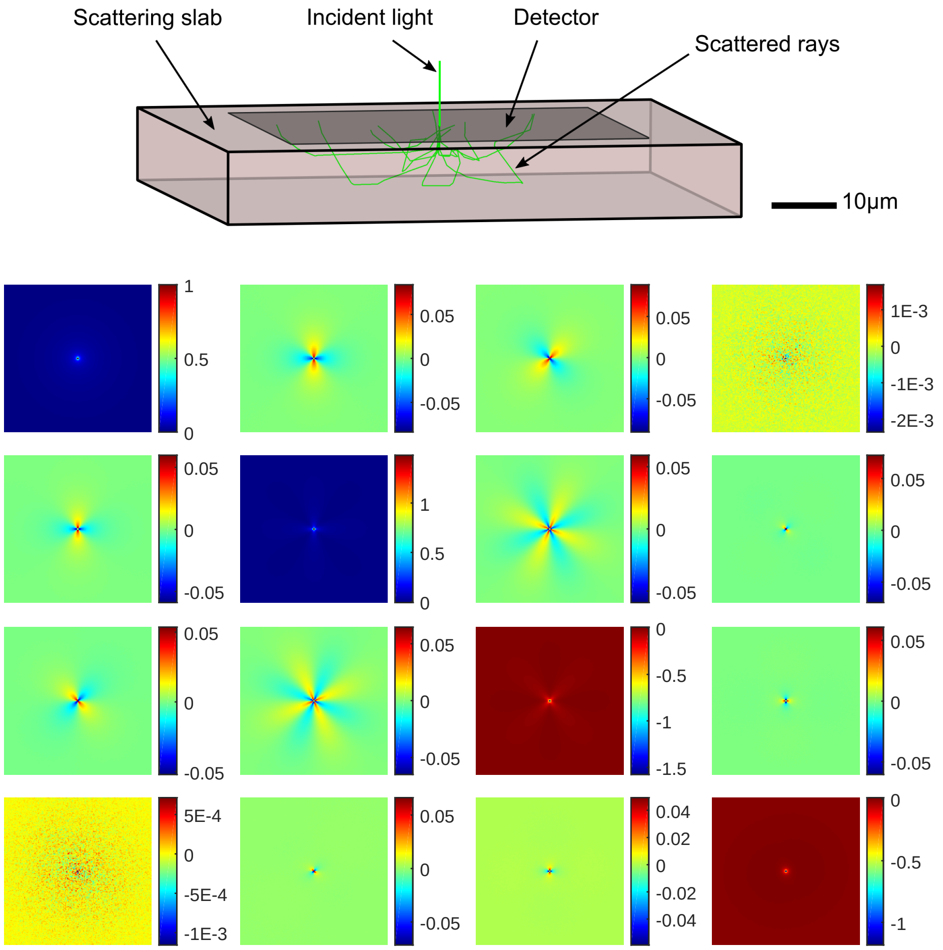

Supplementary Figure 9: Mueller matrices for light backscattered from a scattering slab. Photons are launched into a slab of turbid media, perpendicular to the surface. They propagate and scatter multiple times, and the intensity and polarisation state is recorded by a detector as they exit the surface of the slab on the same side as they entered. The Mueller matrix relates the input and output states of polarisation for each pixel of the detector: the plots show the spatially-resolved Mueller matrices for the spatial locations of the exit points relative to the central entry point, normalised to the central value of the (1,1) plot.

Supplementary Table 1: Optical properties of the retinal layers. Thickness (T), anisotropy factor (g), scattering coefficient (μ_s), and absorption coefficient (μ_a) are shown for wavelengths 532 nm and 633 nm. The parameters are taken from previously published data[11–16], absorption of Choroid was assumed to be that of systemic oxygenated blood.

Layer		$\lambda = 532$nm			$\lambda = 633$nm		
	T (µm)	g	μ_s (mm^{-1})	μ_a (mm^{-1})	g	μ_s (mm^{-1})	μ_a (mm^{-1})
Retina	200	0.97	3.1	0.15	0.97	3.1	0.15
Arterioles	-	0.957	70	23.495	0.979	87.54	0.2874
Venules	-	0.957	70	22.966	0.979	87.54	0.9773
RPE	10	0.84	117	33	0.84	114	18
Choroid	250	0.945	73.1	23.495	0.979	87.54	0.2874
Sclera	700	0.9	102.72	0.46	0.9	85	0.37

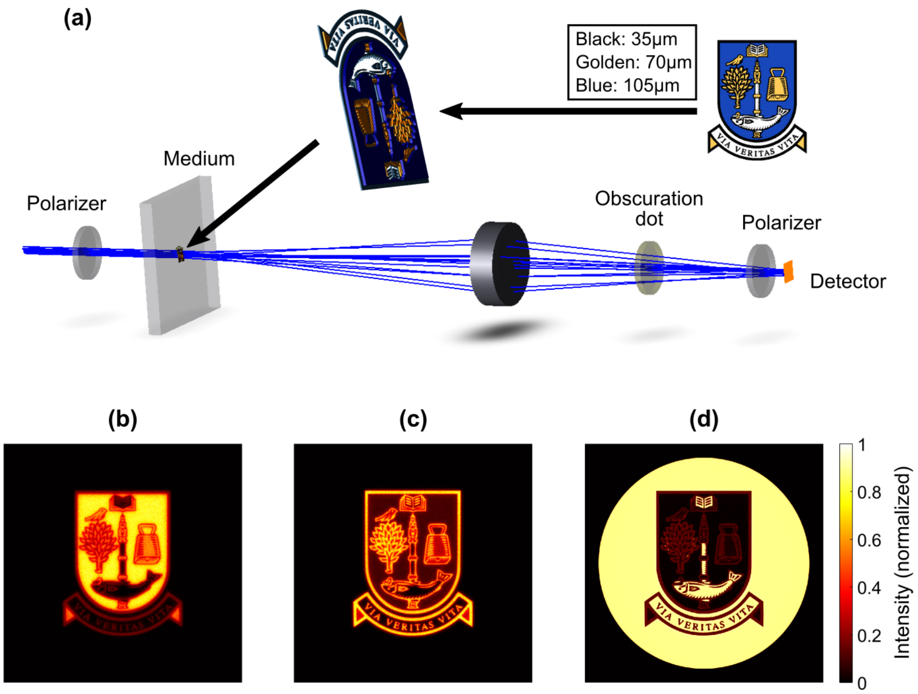

Supplementary Figure 10: Polarimetric imaging experiment. Simulation of an imaging demonstration for Mie scattering within a CAD-defined volume. The setup consists of a collimated beam directed towards a scattering object. A lens collects the scattered light and forms an image of the CAD object at the detector. The CAD object was defined based on the image (University of Glasgow coat of arms) shown in (a), constructed as a volume with various thicknesses: regions in black, gold and blue from the original image were assigned thicknesses of 35 μm, 70 μm and 105 μm respectively. The simulation is performed in three different scenarios with results shown in (b-d): in (b) the illumination and detector polarisers are mutually orthogonal and there is no obscuration dot, tending to preferentially transmit multiply-scattered depolarised light; in (c) a central obscuration was introduced at the lens focal plane to filter out unscattered (ballistic) light but without polarisers; whereas in (d) both the polarisers and obscuration dot are missing and therefore both scattered and unscattered light is detected. For the cases (b) and (c) only light that has been scattered is detected, and in particular the de-polarisation through scattering effect is exploited in (b).

The CAD model of the network of blood vessels was built from a retinal image and the segmentation of the vessels, which are available from a database of fundus images[17]. The aim was to construct a 3D mesh of the vasculature and the main steps to build the CAD model were as follows:

- We started from the binary image corresponding to the segmentation of the vessels.
- A node of the mesh was assigned at the centre of each foreground pixel of the binary image (that is, one node per pixel corresponding to vessels, and excluding background pixels).
- The segmented image was then *skeletonised*: i.e. the width of the segmented vessels was shrunk to one single pixel at the centre of the vessel, to build the *skeleton* image.
- The thickness (diameter) of the vessels was estimated for each location in the *skeleton* image (this was done by calculating the distance in pixels from the pixel in the skeleton to the background).
- For each of the foreground pixels of the initial binary image (segmented vessels), a height was assigned based on the diameter of the vessel and the distance of the pixel to the center of the vessel: $h = \sqrt{D^2/4 - d^2}$ where D is the thickness (diameter) of the vessel and d is the distance of the pixel to the vessel centre. Note that pixels at the centre of the vessel had a height equal to the radius of the vessel, and pixels at the edge of the vessel had a zero height.
- The mesh created up to the previous step corresponds to one-sided "shell" of the vessel, and so the mesh was duplicated with exact but negative heights such that the two meshes formed a closed volume.
- Finally, an additional height was added to each node, taking into account its position within the field-of-view, to reproduce the spherical curvature of the retina.

5 Appraisal of oximetry from SLO images

We report here a simulation experiment to explore how hypoxia (low systemic oxygen saturation levels in blood) affects the contrast of the recorded images. Results are summarised in Supplementary Fig. 11. Simulations were repeated for normoxia and hypoxia oxygenation states, using extinction coefficients for partially oxygenated blood calculated for 98% and 70% oxygen saturation in arteries and veins for normoxia, and 88% and 60% for hypoxia; this corresponds to a 10% reduction in systemic oxygen saturation. In Supplementary Fig. 11 cross-section intensities for two veins and two arteries were selected (at locations labelled ‘A’, ‘B’, ‘C’ and ‘D’), and furthermore the contrast at the arterial location marked with $\star$ was analysed with results shown in the top-right graph. These results show:

- Confocality increases the contrast at 633nm, as absorption is lower (leading to a tissue PSF much larger than the pinhole) and the higher contribution of double-pass light paths is more noticeable. This is apparent in the contrasts calculated at $\star$ and plotted at the bar-graph.
- Fundus reflectivity is higher at red wavelengths but only in non-confocal configurations (cf. first and second rows of graphs. This is due to a lower absorption at these wavelengths but also a wider tissue PSF so that more light is rejected for confocal configurations.
- Contrast is increased with confocality, which is expected as double-pass light paths are more significant; this is most noticeable for the smaller-calibre arteries at $\lambda = 633\,\text{nm}$ (note for instance how arteriole ‘D’ has very low contrast for non-confocal imaging at 633 nm, but exhibits good contrast for the confocal configuration).
- Background reflectivity at 633 nm varies with systemic oxygen saturation level (see first row of graphs), as partial deoxygenation of the choroid increases absorption.

Secondly, in Supplementary Fig. 12 we show an example of retinal oximetry. Several vessels were tracked and labelled (veins/arteries), as shown in the top-left image: veins and arteries are highlighted with blue and red lines respectively. Using images recorded at 633 nm and 532 nm the Optical Density Ratio (ODR) at each vessel location was calculated, and oxygen saturation along the vessels measured. The images in Supplementary Fig. 12 were recorded using a pinhole diameter of 300 µm. Vessels were tracked using a standard semi-automated vessel-tracking algorithm that provided locations of the vessel centre path. Intensity along the vessel paths were then extracted from the images, and a median filter with 15 pixels width was applied

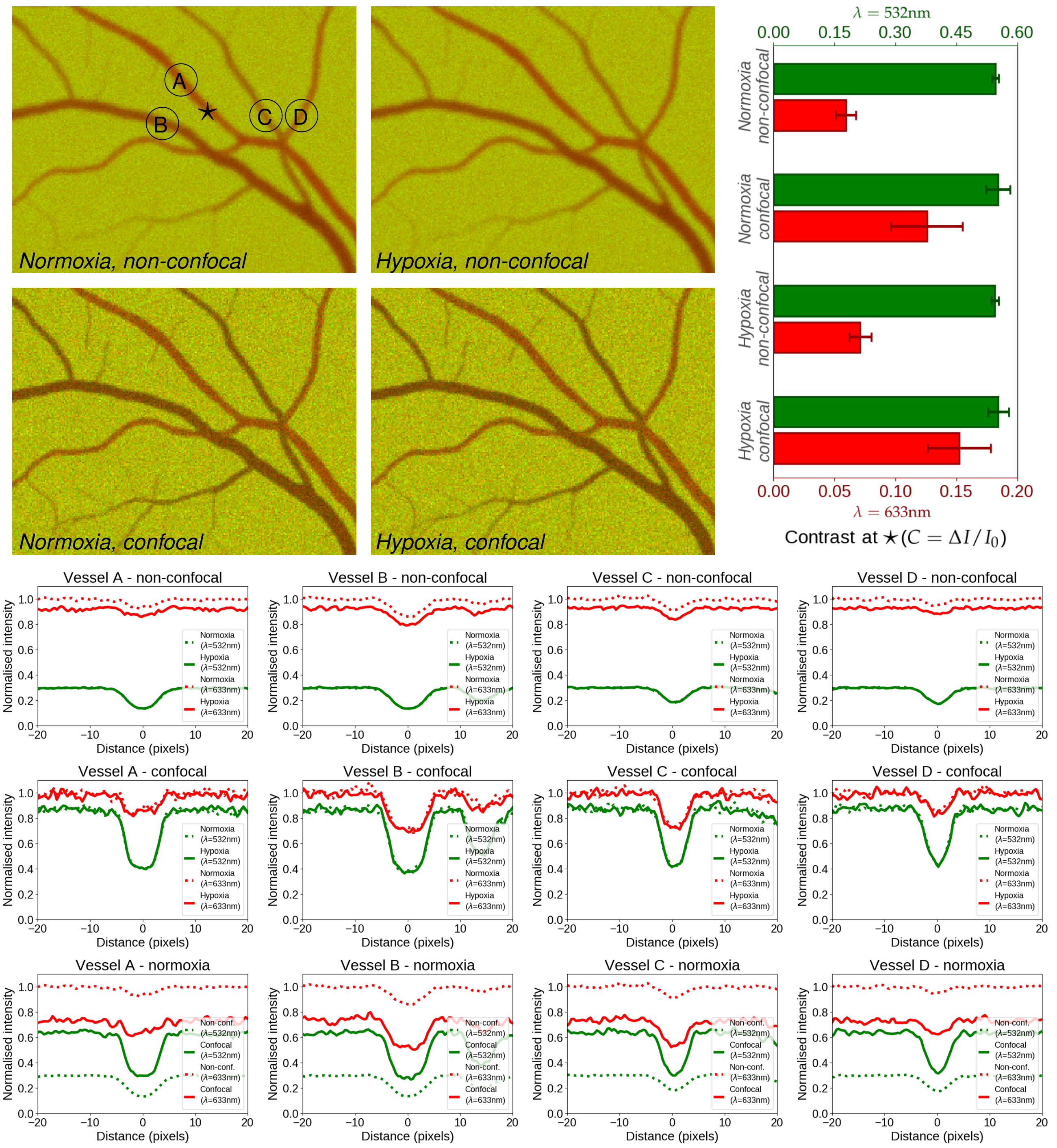

Supplementary Figure 11: Oximetric data acquisition with the SLO. Non-confocal ($D = 1000\,\mu$m) and confocal ($D = 100\,\mu$m) images with normoxia and hypoxia (systemic arterial and venous oxygenation both reduced by 10%). The pseudo-colour images are composed by mapping the $\lambda = 633$ nm image onto the red channel and the $\lambda = 532$ nm onto the green channel. The graphs show the intensity profiles through the cross-section of the vessels labelled 'A', 'B', 'C' and 'D' and the top-right bar-graph shows the calculated contrast of the artery at location labelled with ★ (contrast was calculated as $C = \Delta I / I_0$ where I_0 is the intensity in the region without vessel and ΔI is the intensity drop at the vessel centre; intensities with and without vessel where calculated by tracing 10^7 rays, and the measure was repeated 20 times to determine mean and standard deviation).

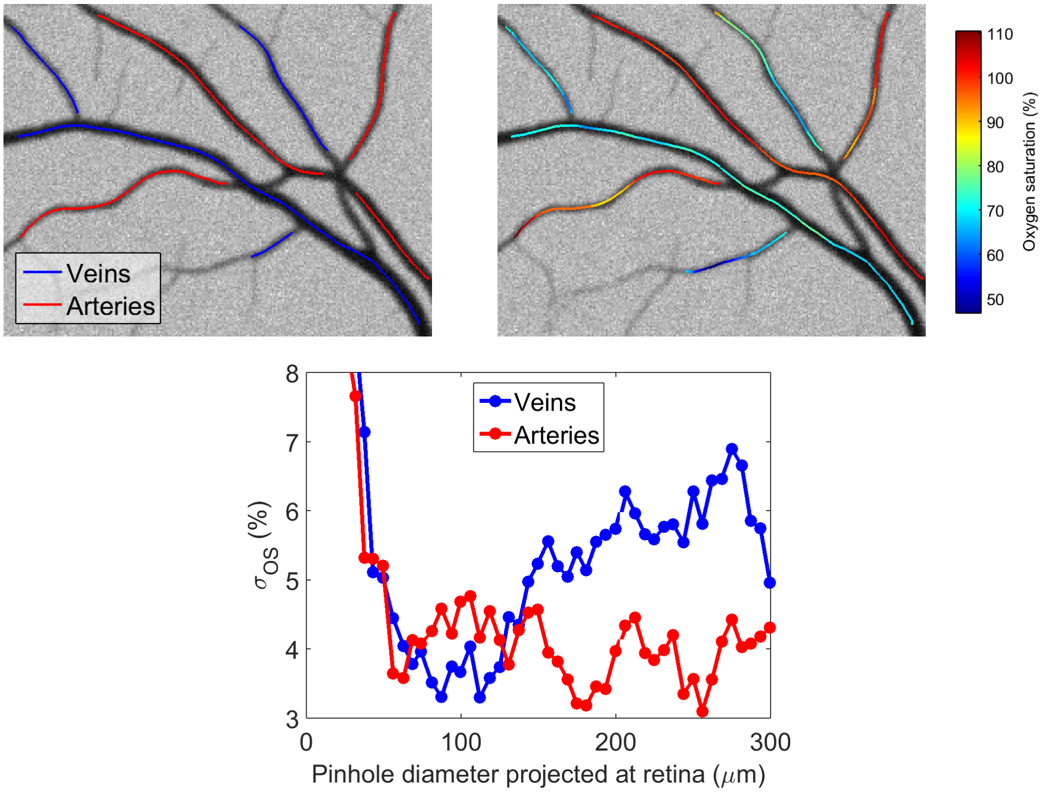

Supplementary Figure 12: Oximetry calculation. Top image shows tracked vessels with ground-truth oxygenation; ODRs were calculated and the groundtruth was used for calibration. Middle image shows calculated oximetry. The process was repeated for different pinhole sizes and the standard deviation of errors at each pixel against ground truth is plotted in the bottom graph, suggesting the expected trade off between higher confocality and reduced signal-to-noise ratio.

to smooth the profile and suppress outliers. Next, the background intensity, I_0, was measured by averaging the intensity at a region with no vessels, and the optical density at each wavelength was computed by[2],

$$\mathrm{OD}_{\lambda} = \log_{10}\left(\frac{I_v}{I_0}\right) \tag{2}$$

where I_v is the intensity at the centre of the vessel. Next, the Optical Density Ratio,

$$\mathrm{ODR} = \frac{\mathrm{OD}_{\lambda=633\,\mathrm{nm}}}{\mathrm{OD}_{\lambda=532\,\mathrm{nm}}} \tag{3}$$

was calculated, and again filtered with a median filter with 15 pixels width.

If one isobestic wavelength is used (that is, the extinction coefficient is not sensitive to oxygen saturation) the ODR exhibits a linear variation with oxygen saturation[18],

$$\mathrm{OS} = \mathrm{ODR}\cdot\beta_1 + \beta_2 \tag{4}$$

where OS is oxygen saturation, and $\beta_{1,2}$ are constants that are calculated by calibration,

$$\beta_1 = \frac{\mathrm{OS}_{\mathrm{arteries}} - \mathrm{OS}_{\mathrm{veins}}}{\overline{\mathrm{ODR}}_{\mathrm{arteries}} - \overline{\mathrm{ODR}}_{\mathrm{veins}}} \tag{5}$$

$$\beta_2 = \frac{\mathrm{OS}_{\mathrm{arteries}}\cdot\mathrm{ODR}_{\mathrm{veins}} - \mathrm{OS}_{\mathrm{veins}}\cdot\mathrm{ODR}_{\mathrm{arteries}}}{\overline{\mathrm{ODR}}_{\mathrm{veins}} - \overline{\mathrm{ODR}}_{\mathrm{arteries}}} \tag{6}$$

where the overscore refer to the mean of the ODRs (excluding 15% of highest and lowest values before calculating the arithmetic mean of the 70% remaining values); veins and arteries had known oxygen saturation values of $\mathrm{OS}_{\mathrm{veins}} = 70\%$ and $\mathrm{OS}_{\mathrm{arteries}} = 98\%$ respectively. Oxygen saturation maps for the tracked vessels were computed and plotted in Supplementary Fig. 12.

To explore how confocality (as determined by pinhole size) affects accuracy of oximetry, calibration and computation of the oxygen saturation maps were repeated for a range of pinhole sizes. In each case the error (deviation of calculated OS from ground truth) was calculated, and the standard deviation for all locations along the tracked vessels is plotted in Supplementary Fig. 12 as a function of pinhole size. These results quantify the expected trend: for large pinholes, the contrast of the vessels is lower, reducing the responsivity of the ODR to oxygen saturation, so that small errors in the measurement of ODR lead to large errors in calculated OS. Conversely, decreasing the pinhole size leads to higher vascular contrast and hence higher responsivity of ODR to oxygenation, but the associated lower light levels yield a lower detected signal-to-noise ratio, which tends to increase errors in calculated OS as the pinhole size decreases. Consequently, there is an optimum trade of these two effects, for which the accuracy and precision of the oximetry is highest. From Supplementary Fig. 12, it can be determined that for arteries the optimum diameter of the pinhole (projected onto the retina) is greater than 60 µm, while for veins it is between about 70 and 120 µm: that is a pinhole with a diameter of between about 70 and 120 µm is approximately optimal for oximetry and for general high-contrast imaging of the vasculature. Our tool therefore provides for the first time, the capability to predict and optimise ophthalmic instruments, such as a SLO, as a function of parameters, such as systemic and vascular oxygenation, retinal pigmentation, vessel calibre, optical illumination and optical aberrations within the ophthalmoscope. For example, the impact on image contrast of optical aberrations and polarisation variations, such as can exist for large field angles, can be predicted with higher accuracy.

References

1. Campbell, F. W. & Gregory, A. H. Effect of size of pupil on visual acuity. *Nature* **187**, 1121–1123 (1960).
2. Beach, J. M., Schwenzer, K. J., Srinivas, S., Kim, D. & Tiedeman, J. S. Oximetry of retinal vessels by dual-wavelength imaging: calibration and influence of pigmentation. *J. Appl. Physiol.* **86**, 748–758 (1999).
3. Smith, M. H., Denninghoff, K. R., Lompado, A. & Hillman, L. W. Effect of multiple light paths on retinal vessel oximetry. *Appl. Opt.* **39**, 1183–1193 (2000).

4. Cimalla, P., Walther, J., Mittasch, M. & Koch, E. Shear flow-induced optical inhomogeneity of blood assessed *in vivo* and *in vitro* by spectral domain optical coherence tomography in the 1.3μm wavelength range. *J. Biomed. Opt.* **16**, 116020 (2011).

5. Carles, G., Muyo, G., van Hemert, J. & Harvey, A. R. Combined high contrast and wide field of view in the scanning laser ophthalmoscope through dual detection of light paths. *J. Biomed. Opt.* **22**, 116002 (2017).

6. Dogariu, M. & Asakura, T. Polarization-dependent backscattering patterns from weakly scattering media. *J. Opt.* **24**, 271 (1993).

7. Cameron, B. D. *et al.* Measurement and calculation of the two-dimensional backscattering mueller matrix of a turbid medium. *Opt. Lett.* **23**, 485–487 (1998). *Erratum-ibid*, **23**, 1630–1630 (1998).

8. Xu, M. Electric field monte carlo simulation of polarized light propagation in turbid media. *Opt. Express* **12**, 6530–6539 (2004).

9. Bartel, S. & Hielscher, A. H. Monte carlo simulations of the diffuse backscattering mueller matrix for highly scattering media. *Appl. Opt.* **39**, 1580–1588 (2000).

10. Ramella-Roman, J. C., Prahl, S. A. & Jacques, S. L. Three monte carlo programs of polarized light transport into scattering media: part II. *Opt. Express* **13**, 10392–10405 (2005).

11. Liu, W., Jiao, S. & Zhang, H. F. Accuracy of retinal oximetry: a monte carlo investigation. *J. Biomed. Opt.* **18**, 066003 (2013).

12. Prahl, S. Optical absorption of hemoglobin. http://omlc.ogi.edu/spectra/hemoglobin/.

13. Friebel, M., Helfmann, J., Netz, U. J. & Meinke, M. C. Influence of oxygen saturation on the optical scattering properties of human red blood cells in the spectral range 250 to 2000 nm. *J. Biomed. Opt.* **14**, 034001 (2009).

14. Hammer, M., Roggan, A., Schweitzer, D. & Muller, G. Optical properties of ocular fundus tissues —- an in vitro study using the double-integrating-sphere technique and inverse monte carlo simulation. *Phys. Medicine Biol.* **40**, 963 (1995).

15. Bashkatov, A. N., Genina, E. A., Kochubey, V. I. & Tuchin, V. V. Optical properties of human sclera in spectral range 370–2500 nm. *Opt. Spectrosc.* **109**, 197–204 (2010).

16. Preece, S. J. & Claridge, E. Monte carlo modelling of the spectral reflectance of the human eye. *Phys. Medicine Biol.* **47**, 2863 (2002).

17. Budai, A. *et al.* A public database for the evaluation of fundus image segmentation algorithms. *Investig. Ophthalmol. Vis. Sci.* **52**, 1345 (2011).

18. MacKenzie, L. E. & Harvey, A. R. Oximetry using multispectral imaging: theory and application. *J. Opt.* **20**, 063501 (2018).
