# Supplementary material for: Holistic Monte-Carlo optical modelling of biological imaging
Source: Sci Rep. 2019 Nov 1;9:15832. doi: 10.1038/s41598-019-51850-1 (PMC6825179; doi:10.1038/s41598-019-51850-1)
Supplement: Supplementary file 2 — Supplementary information [file 41598_2019_51850_MOESM2_ESM.pdf]

Holistic Monte-Carlo Optical Modelling (HMCOM)

USER GUIDE

1 Introduction

As discussed in the main paper, it is possible to integrate Monte-Carlo simulation of light scattering in tissue within the framework of optical ray-tracing, enabling Holistic Monte-Carlo Optical Modelling (HMCOM) [1]. Such a framework is provided by many commercially available optical-design software packages, such as *Zemax OpticStudio*. This document provides guidance on the practical implementation of HMCOM within *Zemax OpticStudio* including the installation and use of a set of Dynamic Link Libraries (DLLs), which are available for download [2,3]. These DLLs are necessary to accurately model Mie scattering of polarised light, and provide a means of calculating fluorescence generated from diffracted beams, respectively.

In section 2 the software packages and their installation are summarised. In section 3 the use of *Zemax OpticStudio* to perform HMCOM simulation of complete systems is discussed. In section 4 guidance is provided to reproduce an example that uses fluorescence and scattering (example 1 of paper [1]). And in section 5 guidance is provided to reproduce an example that uses diffraction beam propagation with fluorescence (example 2 of paper [1]).

2 Installation

The software provided is installed as plugin software to *Zemax OpticStudio*. This includes:

- Mie scattering tracking polarisation and generation of fluorescence [2]
- Diffracted beam propagation with fluorescence [3]

The simulation files and descriptions for the examples reported in [1] are available as supplementary material, packed in the file 'simulation_files.zip', including auxiliary files required for executing the models. Two of these examples are reproduced in Sections 4 and 5 below with explanations. The DLLs can be downloaded from [2,3] including user manuals and installation instructions.

3 Holistic design using Monte-Carlo optical ray-tracing

Commercially available optical design software employ optical raytracing to model, and optimise, optical systems. Optical design is a complex process that involves a large number of variables. It uses an approach termed "sequential ray tracing". The approach assumes a sequence of ordered surfaces that are defined from the first surface (which defines the object or the light source) to the last surface (which defines the image plane). Surfaces have a thickness (defining the distance to the next surface) a glass (defining the optical properties of the material between the surface and the next surface), an aperture value, and other parameters that define its shape (such as curvature, etc). These parameters define the optical system completely. To model, analyse or optimise the performance, light rays are traced from the object to the image surfaces, refracting according to Snell's law at each surface. The arrival location and phase of different light rays are recorded at the image surface to calculate a metric of imaging performance.

This sequential mode, is very well suited for the optical design process, but has some limitations. In particular, it cannot model biological microscopy, including the interaction of the light propagation

at the sample with the optics as referred above. This, however, can be overcome by using a so-called non-sequential mode. In this mode, instead of surfaces, 3D objects are defined and located within a coordinate system together with the light sources and detectors. Ray-tracing therefore functions differently from sequential ray-tracing: each ray is generated at the light source and propagated until it interacts with an object (which can be a lens, scattering object, etc) and then to the next object, and so on, until it either misses all objects, encounters an absorbing object, or hits a detector.

To perform non-sequential ray-tracing using *Zemax OpticStudio*, the "Non-Sequential" mode should be used as is shown in Figure 1. In this mode the "Non-sequential Component Editor" is used to insert objects (including light sources and detectors) that define the optical system to be simulated.

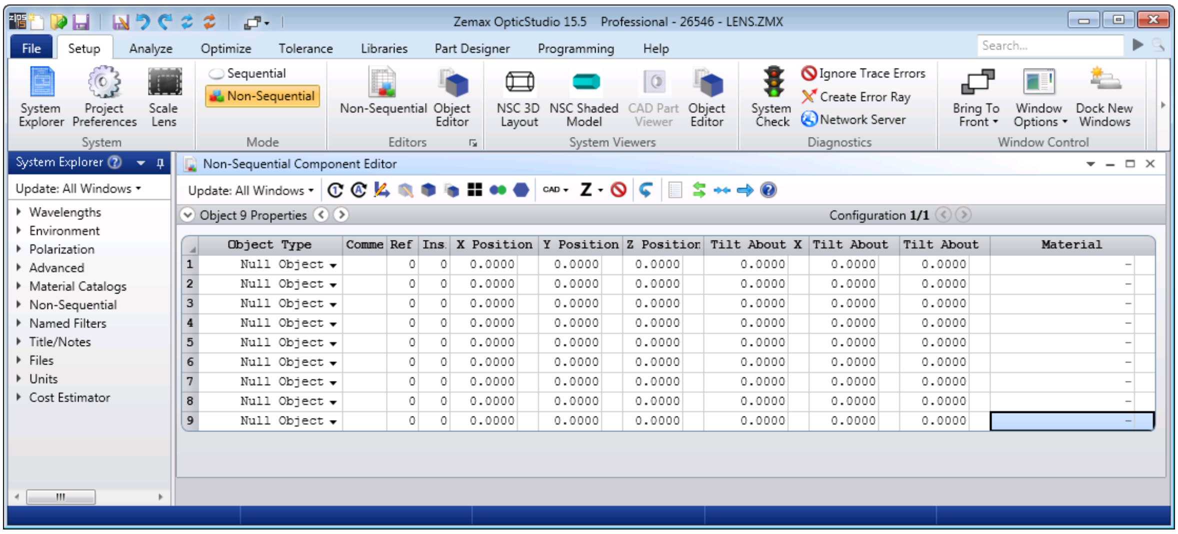

Figure 1: Selection of Non-Sequential mode and view of the Non-Sequential Component Editor where objects, light sources and detectors can be defined.

A system is defined using *Zemax OpticStudio* spreadsheet interface (the "Non-Sequential Component Editor") to locate and define characteristics of objects, detectors and light sources. A Monte-Carlo simulation is executed using the *Ray Trace* tool under the *Analyze* tab as shown in Figure 2. Upon completion, irradiance at a defined detector can be inspected using the *Detector viewer tool*.

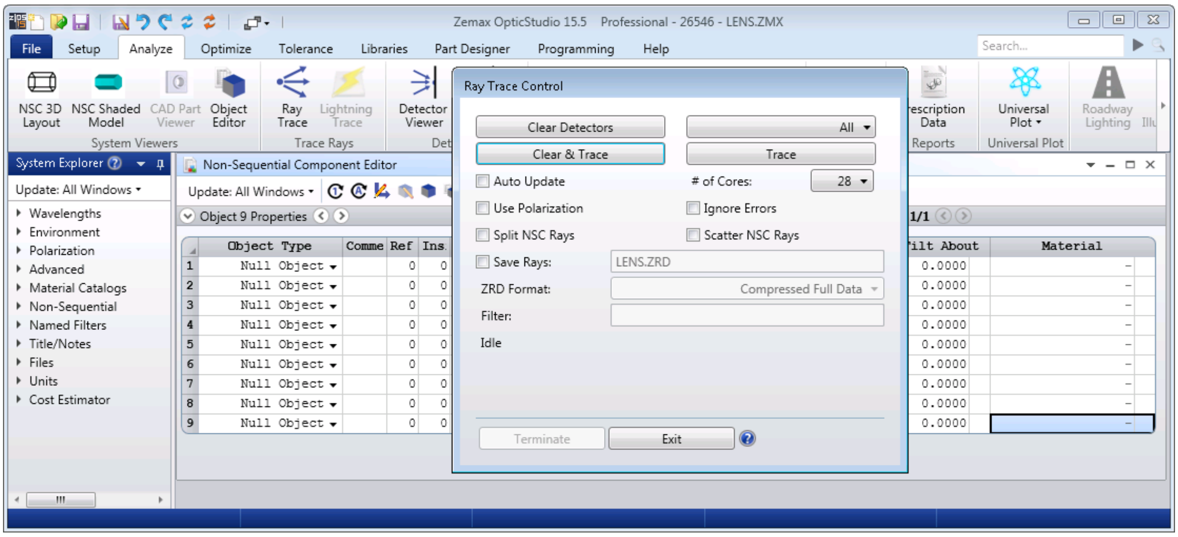

Figure 2: Ray Trace Control tool to clear detectors and perform new ray tracings.

A range of other utilities, and a range of different object properties and shapes are available as described in the *Zemax OpticStudio* user manual.

4 Example A: micro-endoscope

Figure 1 in the main text of [1] shows the HMCOM model used to model microscopy of a labelled neuron using a microendoscope. The HMCOM model is implemented using the file ‘micro_endoscope.zmx’ included within the supplementary material (‘simulation_files.zip’). Opening this file in *Zemax OpticStudio* yields the spreadsheet interface shown in Figure 3, which constructs the 3D arrangement of standard optical elements (lenses, beam-splitters, etc, as shown in the figure) and also a CAD model of the neuron to be imaged. It is also necessary to install the DLL *RayPolMieScattering* [2] for implementation of polarisation-senstive Mie scattering and auxiliary files as described in ‘readme.txt’ included in ‘simulation_files.zip’). Note how, for example, the detector position *Z position* for object 15 can be adjusted to vary the imaging focus. See the *Zemax OpticStudio* user manual for a description of the parameters associated with each object type. The output image, such as shown in Figure 3 is generated by performing a ray-trace.

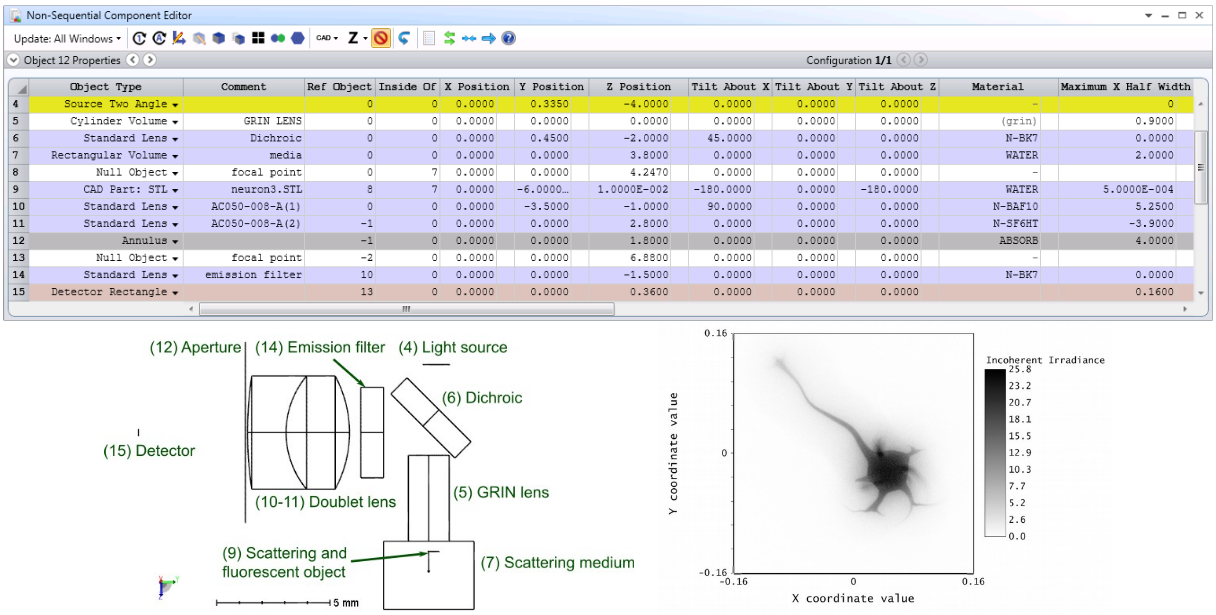
Non-Sequential Component Editor

	Object Type	Comment	Ref Object	Inside Of	X Position	Y Position	Z Position	Tilt About X	Tilt About Y	Tilt About Z	Material	Maximum X Half Width
4	Source Two Angle		0	0	0.0000	0.3350	-4.0000	0.0000	0.0000	0.0000	-	0
5	Cylinder Volume	GRIN LENS	0	0	0.0000	0.0000	0.0000	0.0000	0.0000	0.0000	(grin)	0.9000
6	Standard Lens	Dichroic	0	0	0.0000	0.4500	-2.0000	45.0000	0.0000	0.0000	N-BK7	0.0000
7	Rectangular Volume	media	0	0	0.0000	0.0000	3.8000	0.0000	0.0000	0.0000	WATER	2.0000
8	Null Object	focal point	0	7	0.0000	0.0000	4.2470	0.0000	0.0000	0.0000	-	
9	CAD Part: STL	neuron3.STL	8	7	0.0000	-6.0000…	1.0000E-002	-180.0000	0.0000	-180.0000	WATER	5.0000E-004
10	Standard Lens	AC050-008-A(1)	0	0	0.0000	-3.5000	-1.0000	90.0000	0.0000	0.0000	N-BAF10	5.2500
11	Standard Lens	AC050-008-A(2)	-1	0	0.0000	0.0000	2.8000	0.0000	0.0000	0.0000	N-SF6HT	-3.9000
12	Annulus		-1	0	0.0000	0.0000	1.8000	0.0000	0.0000	0.0000	ABSORB	4.0000
13	Null Object	focal point	-2	0	0.0000	0.0000	6.8800	0.0000	0.0000	0.0000	-	
14	Standard Lens	emission filter	10	0	0.0000	0.0000	-1.5000	0.0000	0.0000	0.0000	N-BK7	0.0000
15	Detector Rectangle		13	0	0.0000	0.0000	0.3600	0.0000	0.0000	0.0000		0.1600

Figure 3: Illustration of definition of the micro-endoscope system in [1] using the Non-Sequential mode. Bottom-left: layout of the system (object numbers match the editor row number). Bottom-right: example detector data after performing a ray-trace.

This example uses the polarisation-sensitive Mie scattering DLL, defined in object 7 within the spreadsheet interface shown in Figure 3 (which is the scattering medium) and object 9 (which is the CAD-defined sample, a neuron). To enable light scattering within the volume of an object, the *Volume Physics* tab under the *Object Properties* window should be used as shown in Figure 4. See “Polarization-sensitive scattering in OpticStudio” [4] for a comprehensive description of the Monte-Carlo modelling of light scattering, and polarisation-sentitive Mie scattering in particular.

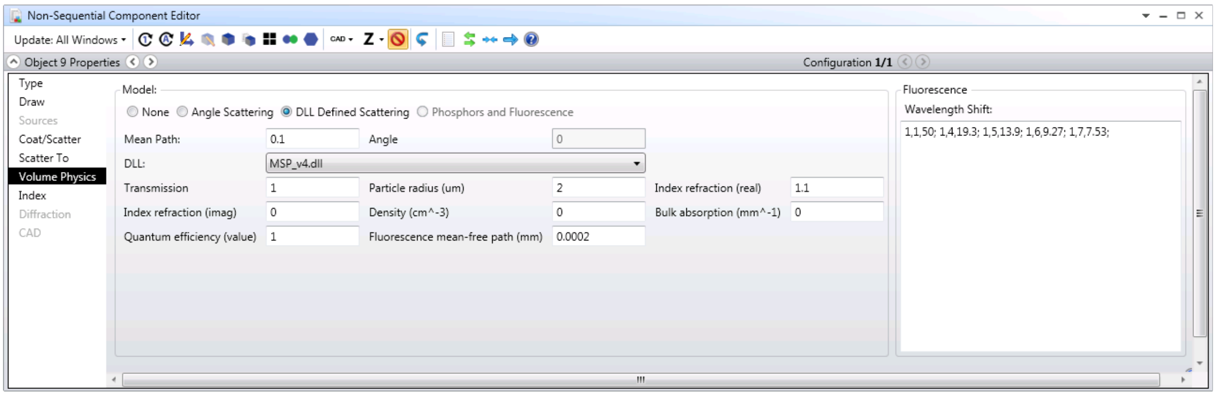

Figure 4: Interface for the MSP DLL, see user manual of the DLL for a description of parameters and further details.

5 Example B: Light-Sheet Fluorescence Microscope

The second example reported in [1] is the simulation of a light-sheet-fluorescence microscope (LSFM). This example shows how to calculate the angular spectrum of a sampled beam, how to use the beam propagation method to propagate the sampled beam, and how to use a light source DLL to generate fluorescence from a sample illuminated by such a beam. The example is executed in three main steps: 1) calculation of the angular spectrum, 2) propagation of a diffracted beam and calculation of fluorescence radiance, and 3) ray-tracing of fluorescence rays.

Step 1: To calculate the angular spectrum, the provided DLL *AngSpecCalc* [3] is used. In the example, this is done to sample the imaging beam of the LSFM, as shown in Figure 5, and can be reproduced by opening the provided file 'LSFM/illumination_arm_absEGFP.zmx' provided in 'simulation_files.zip' (see readme.txt for installation of auxiliary files). By running the simulation the DLL *AngSpecCalc* calculates the electric field defined at the plane where it is located and outputs the data to a file.

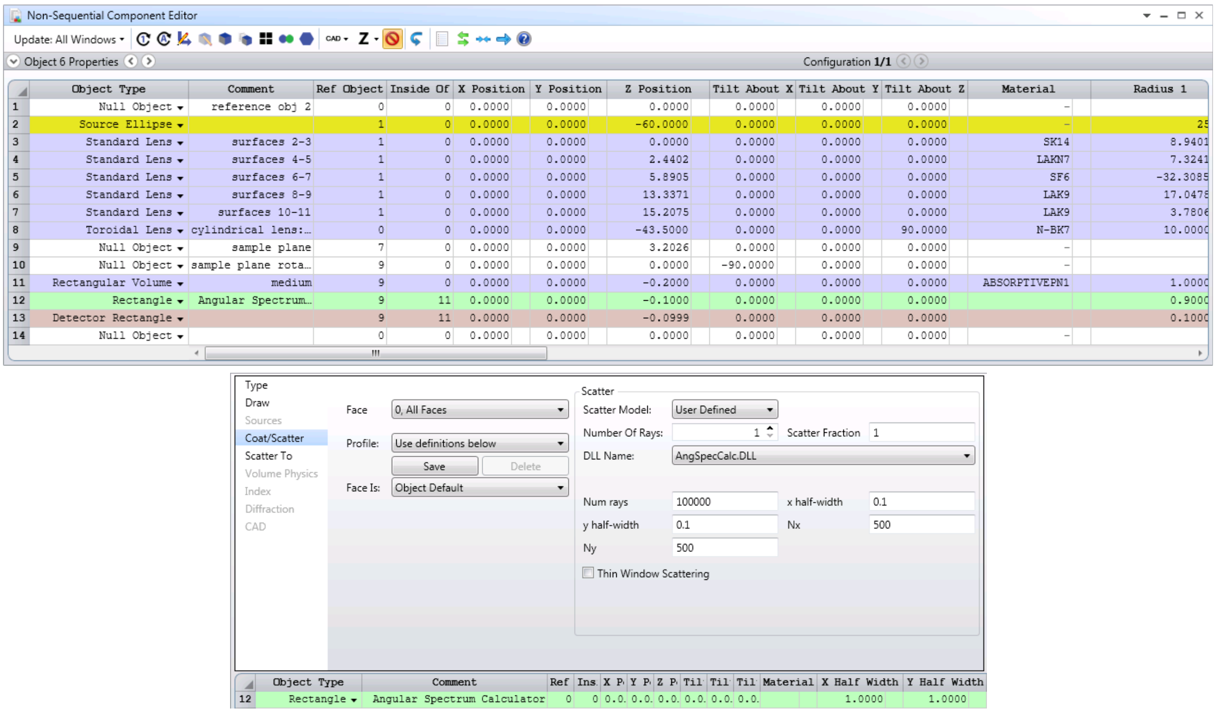

	Object Type	Comment	Ref Object	Inside Of	X Position	Y Position	Z Position	Tilt About X	Tilt About Y	Tilt About Z	Material	Radius 1
1	Null Object	reference obj 2	0	0	0.0000	0.0000	0.0000	0.0000	0.0000	0.0000	-	
2	Source Ellipse		1	0	0.0000	0.0000	-60.0000	0.0000	0.0000	0.0000	-	25
3	Standard Lens	surfaces 2-3	1	0	0.0000	0.0000	0.0000	0.0000	0.0000	0.0000	SK14	8.9401
4	Standard Lens	surfaces 4-5	1	0	0.0000	0.0000	2.4402	0.0000	0.0000	0.0000	LAKN7	7.3241
5	Standard Lens	surfaces 6-7	1	0	0.0000	0.0000	5.8905	0.0000	0.0000	0.0000	SF6	-32.3085
6	Standard Lens	surfaces 8-9	1	0	0.0000	0.0000	13.3371	0.0000	0.0000	0.0000	LAK9	17.0475
7	Standard Lens	surfaces 10-11	1	0	0.0000	0.0000	15.2075	0.0000	0.0000	0.0000	LAK9	3.7806
8	Toroidal Lens	cylindrical lens:...	0	0	0.0000	0.0000	-43.5000	0.0000	0.0000	90.0000	N-BK7	10.0000
9	Null Object	sample plane	7	0	0.0000	0.0000	3.2026	0.0000	0.0000	0.0000	-	
10	Null Object	sample plane rota...	9	0	0.0000	0.0000	0.0000	-90.0000	0.0000	0.0000	-	
11	Rectangular Volume	medium	9	0	0.0000	0.0000	-0.2000	0.0000	0.0000	0.0000	ABSORPTIVEPN1	1.0000
12	Rectangle	Angular Spectrum...	9	11	0.0000	0.0000	-0.1000	0.0000	0.0000	0.0000		0.9000
13	Detector Rectangle		9	11	0.0000	0.0000	-0.0999	0.0000	0.0000	0.0000		0.1000
14	Null Object		0	0	0.0000	0.0000	0.0000	0.0000	0.0000	0.0000	-	

Figure 5: Editor layout for the illumination arm of the LSFM example: object 2 is the illumination light source, objects 3-7 define the microscope objective, object 8 is the cylindrical lens, object 12 is the *AngSpecCalc* DLL, with interface shown below, as a *Coat/Scatter* DLL, see [3] for further details.

Step 2: The second step is the propagation of the beam to calculate the 3D intensity distribution within the sample volume. Example *Matlab* code is provided in [3] to implement the propagation using the beam propagation method, load (or define) the model of the sample (for example from a CAD model; in the provided example the CAD model of the neuron is loaded), voxelise the sample volume, calculate the illumination intensity at the voxelised volume (see paper [1] for details) and save it to a file.

Step 3: The third step is the simulation of imaging. It is based on ray-tracing using *Zemax OpticStudio* and using the DLL *SourceFromDB*, provided in [3]. The DLL imports data generated at the previous step, which defines the generation of fluorescence light rays. In the example this is used with an imaging microscope objective and a detector to simulate image acquisition in a LSFM, as is shown in Figure 6, and is reproduced using the file 'LSFM/imaging_arm_absEGFP.zmx' provided in 'simulation_files.zip' (see 'readme.txt' for installation of auxiliary files).

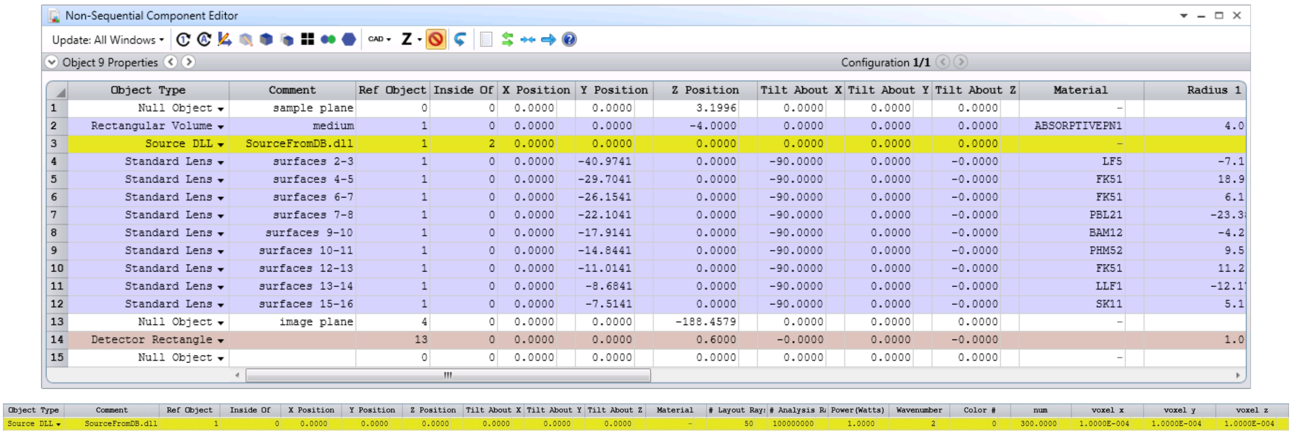

	Object Type	Comment	Ref Object	Inside Of	X Position	Y Position	Z Position	Tilt About X	Tilt About Y	Tilt About Z	Material	Radius 1
1	Null Object	sample plane	0	0	0.0000	0.0000	3.1996	0.0000	0.0000	0.0000	-	
2	Rectangular Volume	medium	1	0	0.0000	0.0000	-4.0000	0.0000	0.0000	0.0000	ABSORPTIVEPN1	4.0
3	Source DLL	SourceFromDB.dll	1	2	0.0000	0.0000	0.0000	0.0000	0.0000	0.0000	-	
4	Standard Lens	surfaces 2-3	1	0	0.0000	-40.9741	0.0000	-90.0000	0.0000	-0.0000	LF5	-7.1
5	Standard Lens	surfaces 4-5	1	0	0.0000	-29.7041	0.0000	-90.0000	0.0000	-0.0000	FK51	18.9
6	Standard Lens	surfaces 6-7	1	0	0.0000	-26.1541	0.0000	-90.0000	0.0000	-0.0000	FK51	6.1
7	Standard Lens	surfaces 7-8	1	0	0.0000	-22.1041	0.0000	-90.0000	0.0000	-0.0000	PBL21	-23.3
8	Standard Lens	surfaces 9-10	1	0	0.0000	-17.9141	0.0000	-90.0000	0.0000	-0.0000	BAM12	-4.2
9	Standard Lens	surfaces 10-11	1	0	0.0000	-14.8441	0.0000	-90.0000	0.0000	-0.0000	PHM52	9.5
10	Standard Lens	surfaces 12-13	1	0	0.0000	-11.0141	0.0000	-90.0000	0.0000	-0.0000	FK51	11.2
11	Standard Lens	surfaces 13-14	1	0	0.0000	-8.6841	0.0000	-90.0000	0.0000	-0.0000	LLF1	-12.1
12	Standard Lens	surfaces 15-16	1	0	0.0000	-7.5141	0.0000	-90.0000	0.0000	-0.0000	SK11	5.1
13	Null Object	image plane	4	0	0.0000	0.0000	-188.4579	0.0000	0.0000	0.0000	-	
14	Detector Rectangle		13	0	0.0000	0.0000	0.6000	-0.0000	0.0000	-0.0000		1.0
15	Null Object		0	0	0.0000	0.0000	0.0000	0.0000	0.0000	0.0000	-	

Object Type	Comment	Ref Object	Inside Of	X Position	Y Position	Z Position	Tilt About X	Tilt About Y	Tilt About Z	Material	# Layout Ray:	# Analysis R:	Power(Watts)	Wavenumber	Color #	num	voxel x	voxel y	voxel z
Source DLL	SourceFromDB.dll	1	0	0.0000	0.0000	0.0000	0.0000	0.0000	0.0000	-	50	100000000	1.0000	2	0	300.0000	1.0000E-004	1.0000E-004	1.0000E-004

Figure 6: Editor layout for the imaging arm of the LFSM example: object 2 is the medium where the sample is located, which is simulated by object 3 that launches fluorescence rays as calculated in step 2; objects 4-12 define the imaging microscope objective and object 14 is the detector. The row in the bottom image is a view of the parameters (interface) of the *SourceFromDB* DLL, implemented as a user-defined light source, see user manual of the DLL in [3] for a description of parameters and further details

The output of this example is the image data at the detector of the imaging arm model. For simulation of a complete LSFM scanning process, the location of the sample (and optionally of the surrounding medium, if scattering is to be included) can be changed in step 1. Steps 1 and 2 need to be repeated for each scanning step.

References

[1] G. Carles, P. Zammit, A. Harvey, "Holistic Monte-Carlo optical modelling of biological imaging"

[2] https://github.com/gcarles/RayPolMieScattering

[3] https://github.com/gcarles/Zemax-DiffractionBeamFluorescence

[4] G. Carles, "Polarization-sensitive scattering in OpticStudio" Zemax Knowledge Base article, https://customers.zemax.com/os/resources/learn/knowledgebase/polarization-sensitive-scattering-in-opticstudio
