# Supplementary figures and images for: Holistic Monte-Carlo optical modelling of biological imaging
Source: Sci Rep. 2019 Nov 1;9:15832. doi: 10.1038/s41598-019-51850-1 (PMC6825179; doi:10.1038/s41598-019-51850-1)

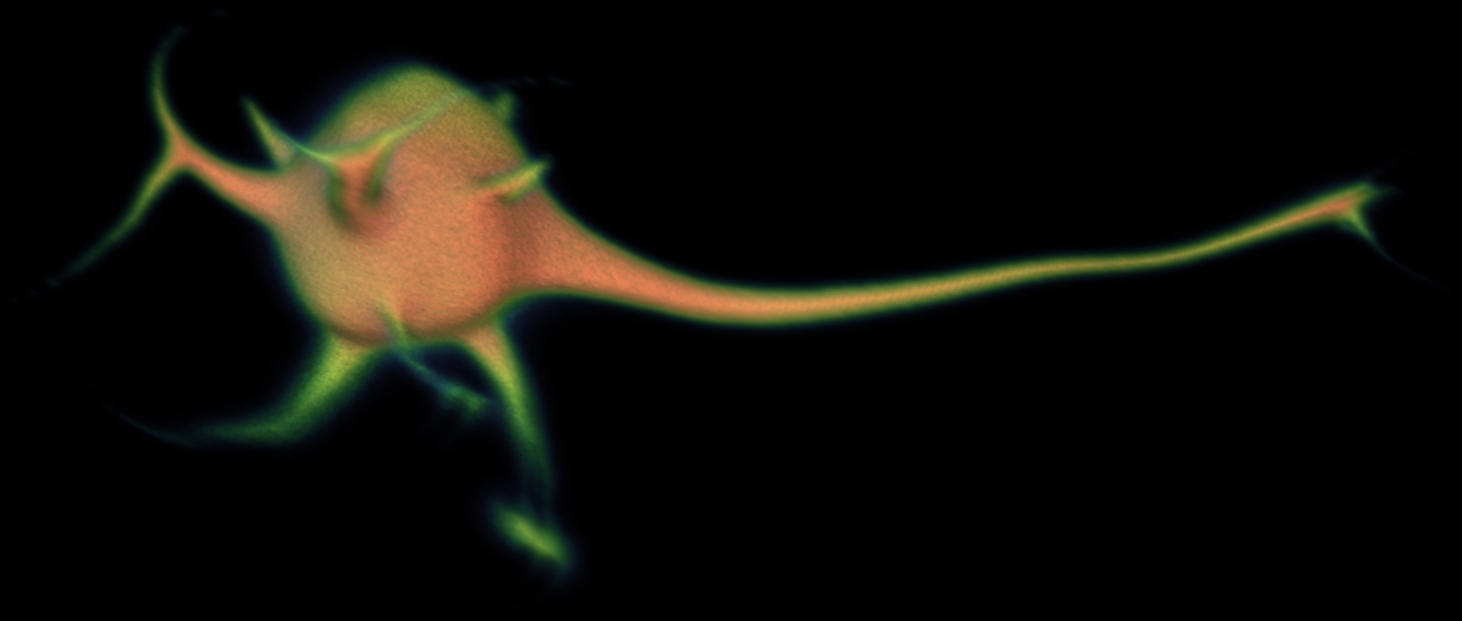

Supplement: Supplementary file 3 — Supplementary information [file 41598_2019_51850_MOESM3_ESM.zip › LSFM/screenshot_lightsheet_3Dreconstruction_noscattering.png]

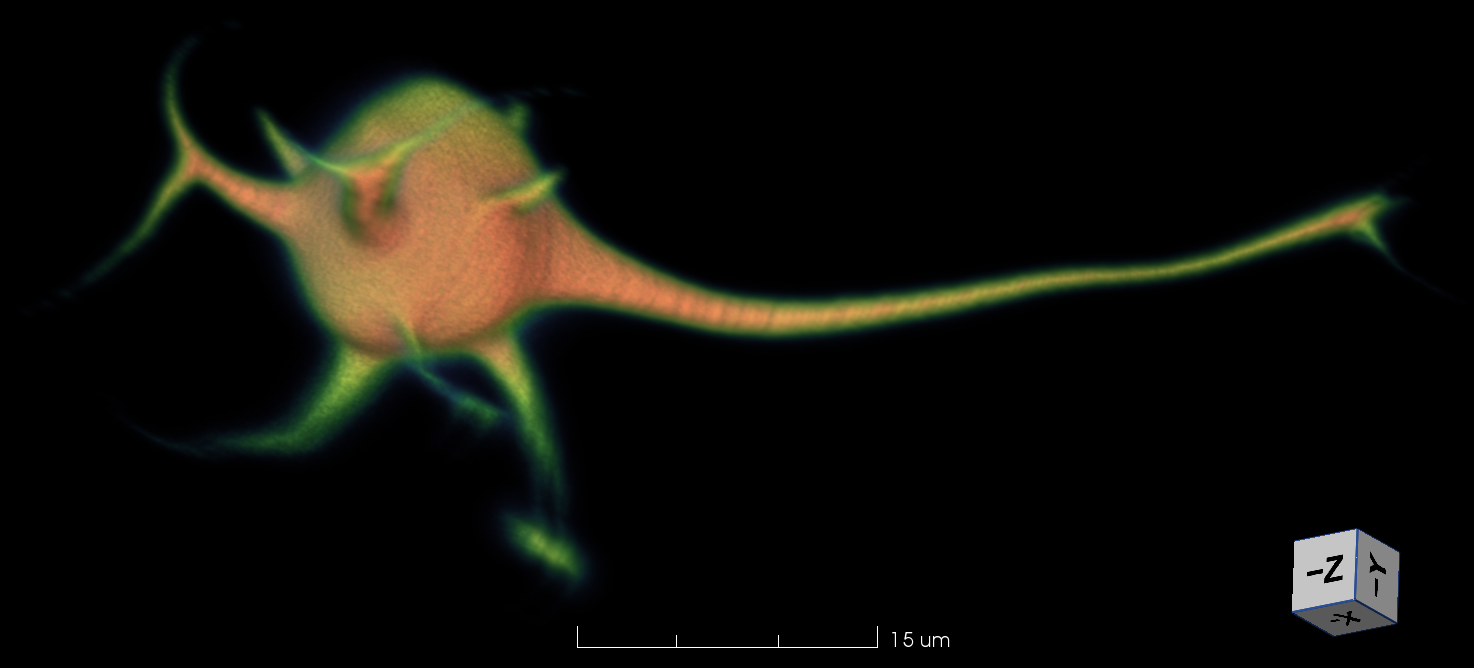

Supplement: Supplementary file 3 — Supplementary information [file 41598_2019_51850_MOESM3_ESM.zip › LSFM/screenshot_lightsheet_3Dreconstruction_scattering.png]

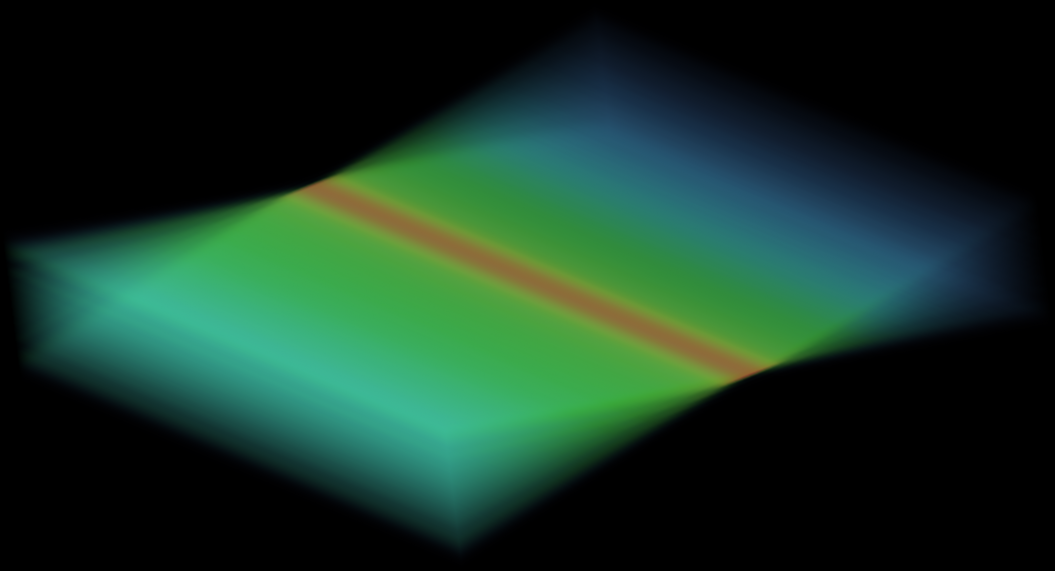

Supplement: Supplementary file 3 — Supplementary information [file 41598_2019_51850_MOESM3_ESM.zip › LSFM/screenshot_lightsheet_BPM_noscattering.png]

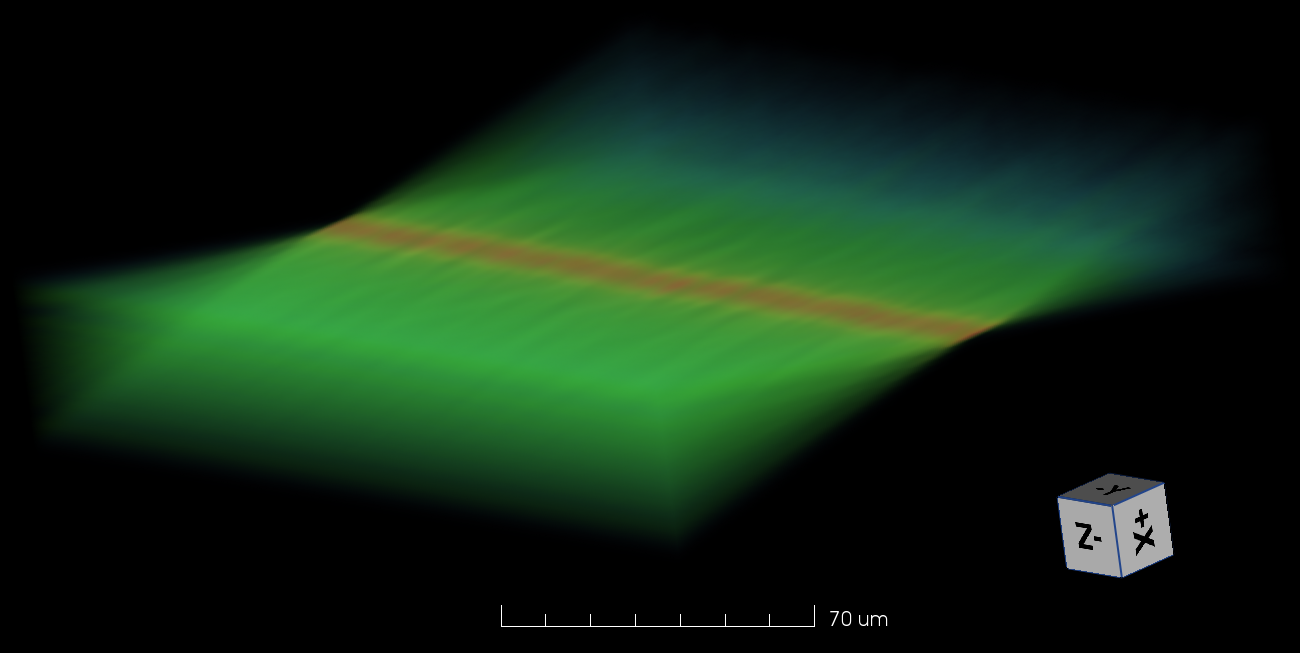

Supplement: Supplementary file 3 — Supplementary information [file 41598_2019_51850_MOESM3_ESM.zip › LSFM/screenshot_lightsheet_BPM_scattering.png]

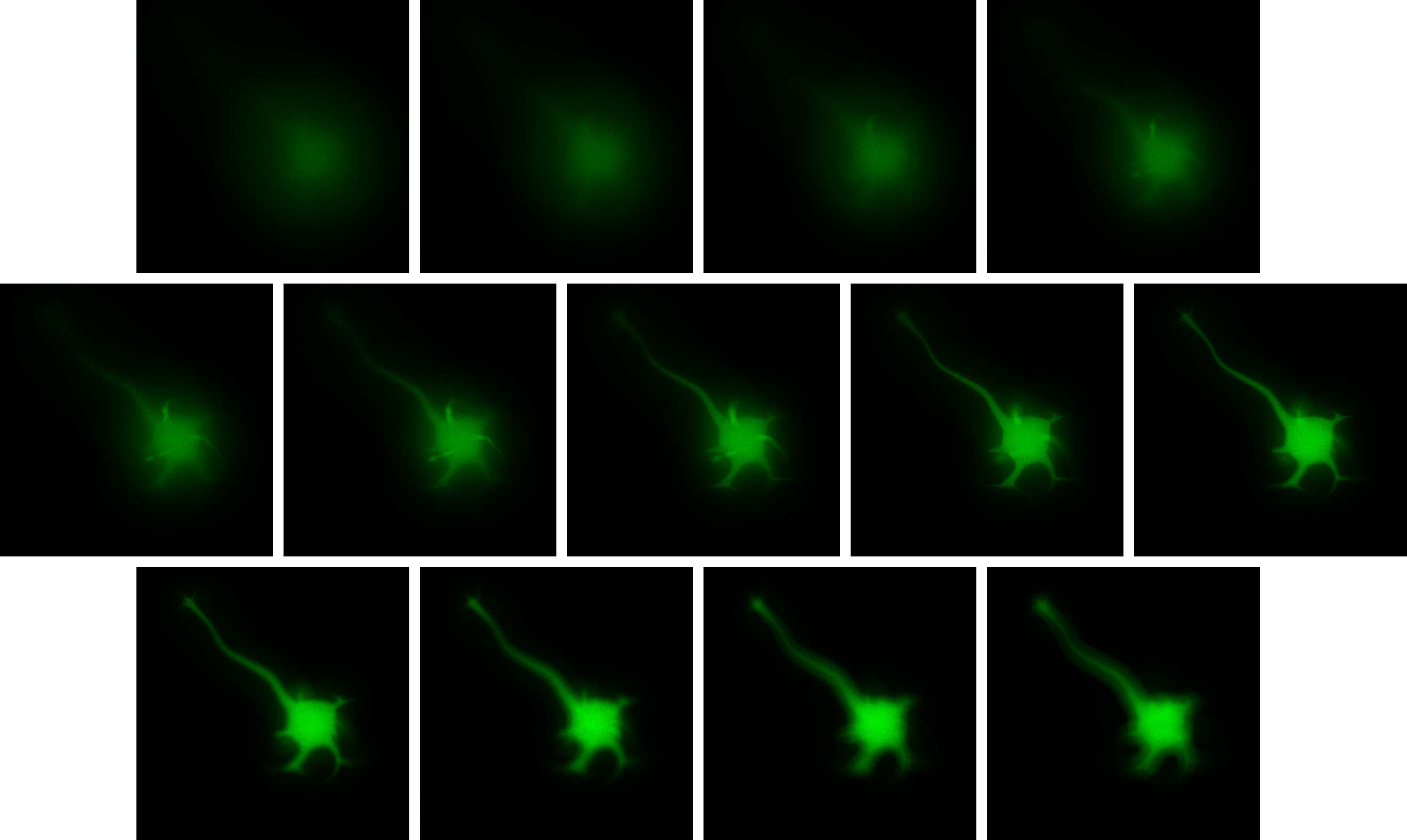

Supplement: Supplementary file 3 — Supplementary information [file 41598_2019_51850_MOESM3_ESM.zip › Microendoscope/img_fig_dof.png]

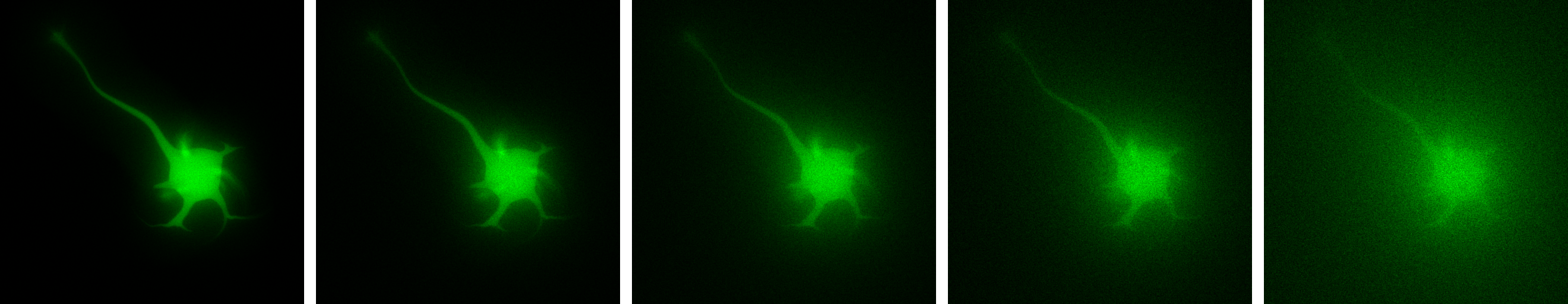

Supplement: Supplementary file 3 — Supplementary information [file 41598_2019_51850_MOESM3_ESM.zip › Microendoscope/img_fig_mus.png]

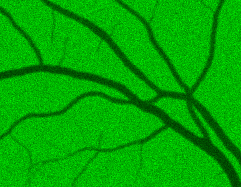

Supplement: Supplementary file 3 — Supplementary information [file 41598_2019_51850_MOESM3_ESM.zip › SLO/img_patch_green_conff5.png]

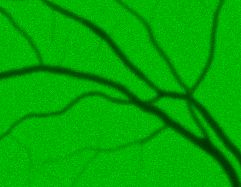

Supplement: Supplementary file 3 — Supplementary information [file 41598_2019_51850_MOESM3_ESM.zip › SLO/img_patch_green_conff50.png]

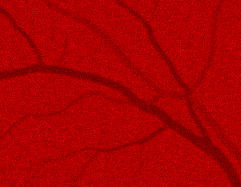

Supplement: Supplementary file 3 — Supplementary information [file 41598_2019_51850_MOESM3_ESM.zip › SLO/img_patch_red_conff5.png]

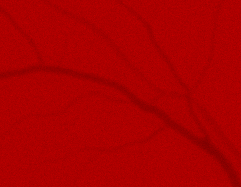

Supplement: Supplementary file 3 — Supplementary information [file 41598_2019_51850_MOESM3_ESM.zip › SLO/img_patch_red_conff50.png]

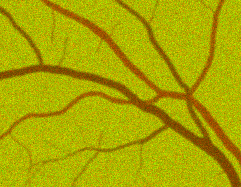

Supplement: Supplementary file 3 — Supplementary information [file 41598_2019_51850_MOESM3_ESM.zip › SLO/img_patch_rgb_conff5.png]

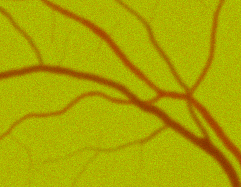

Supplement: Supplementary file 3 — Supplementary information [file 41598_2019_51850_MOESM3_ESM.zip › SLO/img_patch_rgb_conff50.png]

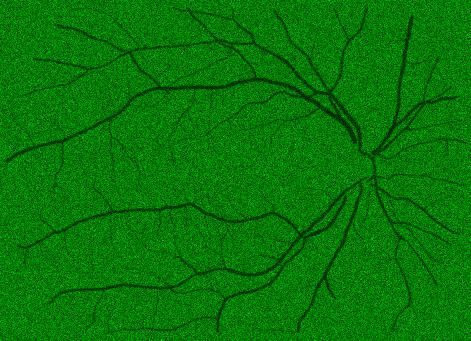

Supplement: Supplementary file 3 — Supplementary information [file 41598_2019_51850_MOESM3_ESM.zip › SLO/img_wide_green_conff5.png]

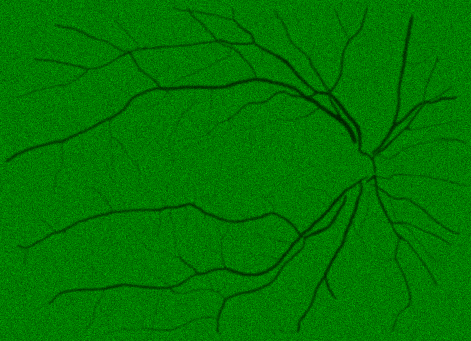

Supplement: Supplementary file 3 — Supplementary information [file 41598_2019_51850_MOESM3_ESM.zip › SLO/img_wide_green_conff50.png]

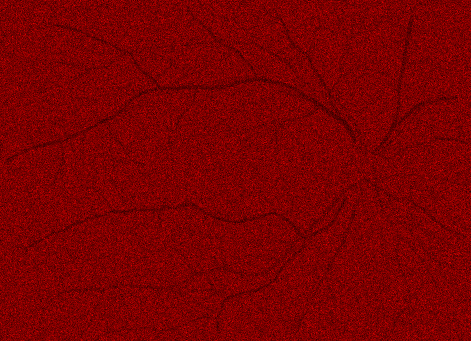

Supplement: Supplementary file 3 — Supplementary information [file 41598_2019_51850_MOESM3_ESM.zip › SLO/img_wide_red_conff5.png]

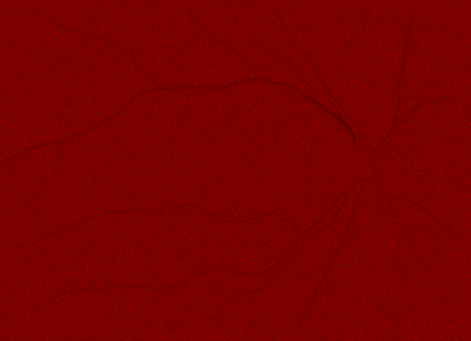

Supplement: Supplementary file 3 — Supplementary information [file 41598_2019_51850_MOESM3_ESM.zip › SLO/img_wide_red_conff50.png]

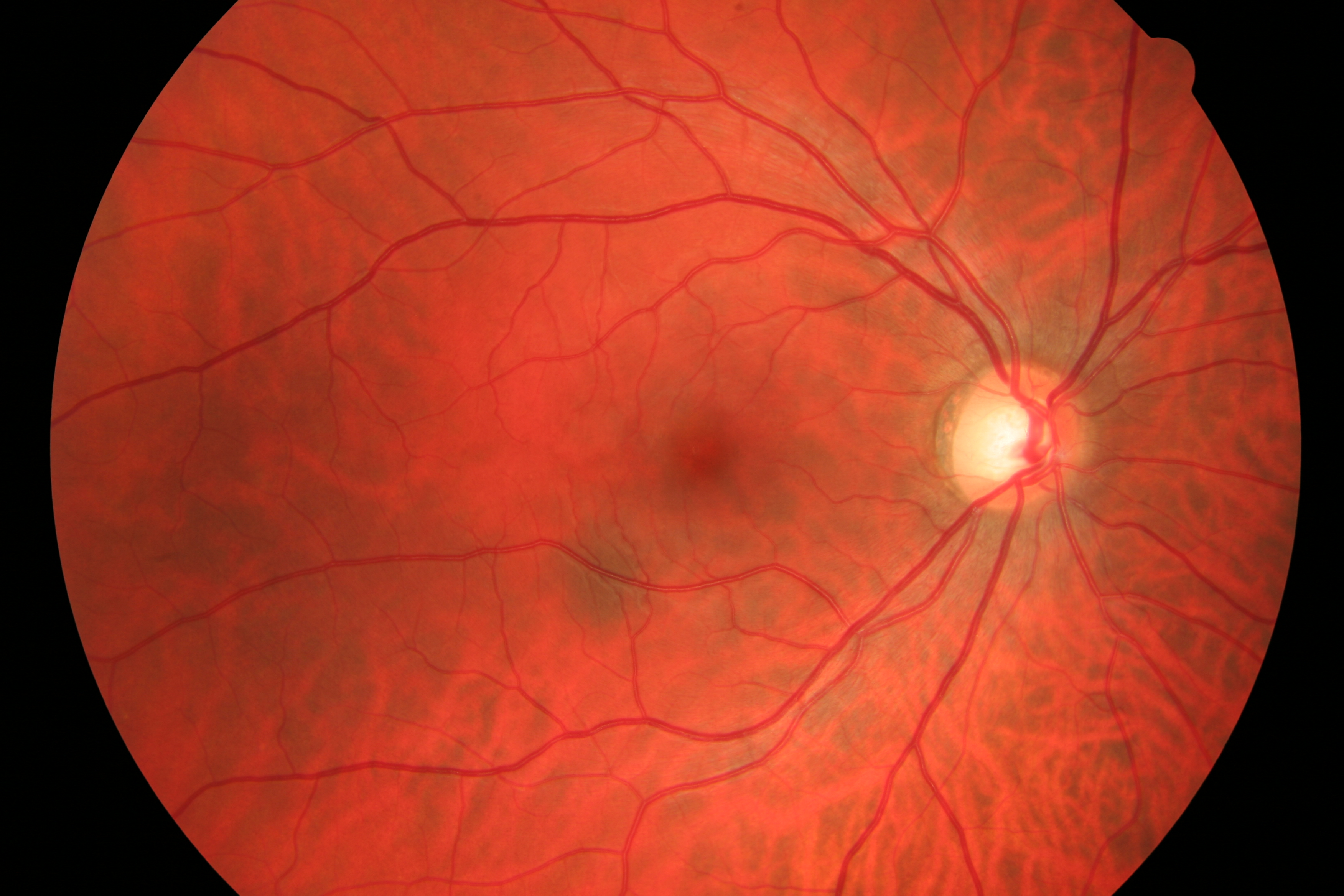

Supplement: Supplementary file 3 — Supplementary information [file 41598_2019_51850_MOESM3_ESM.zip › SLO/vessels_01_h.jpg]

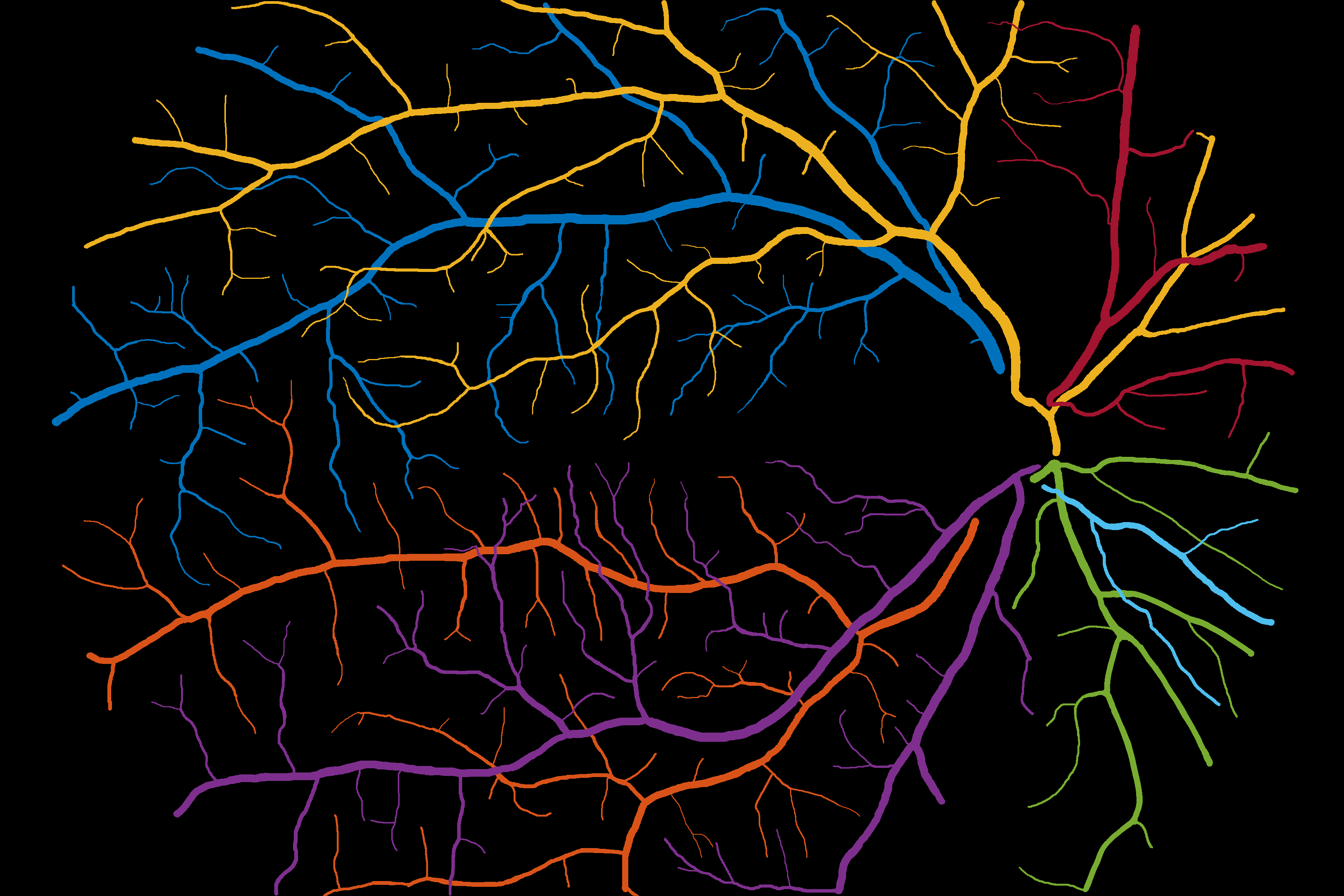

Supplement: Supplementary file 3 — Supplementary information [file 41598_2019_51850_MOESM3_ESM.zip › SLO/vessels_01_h_7networks1.png]

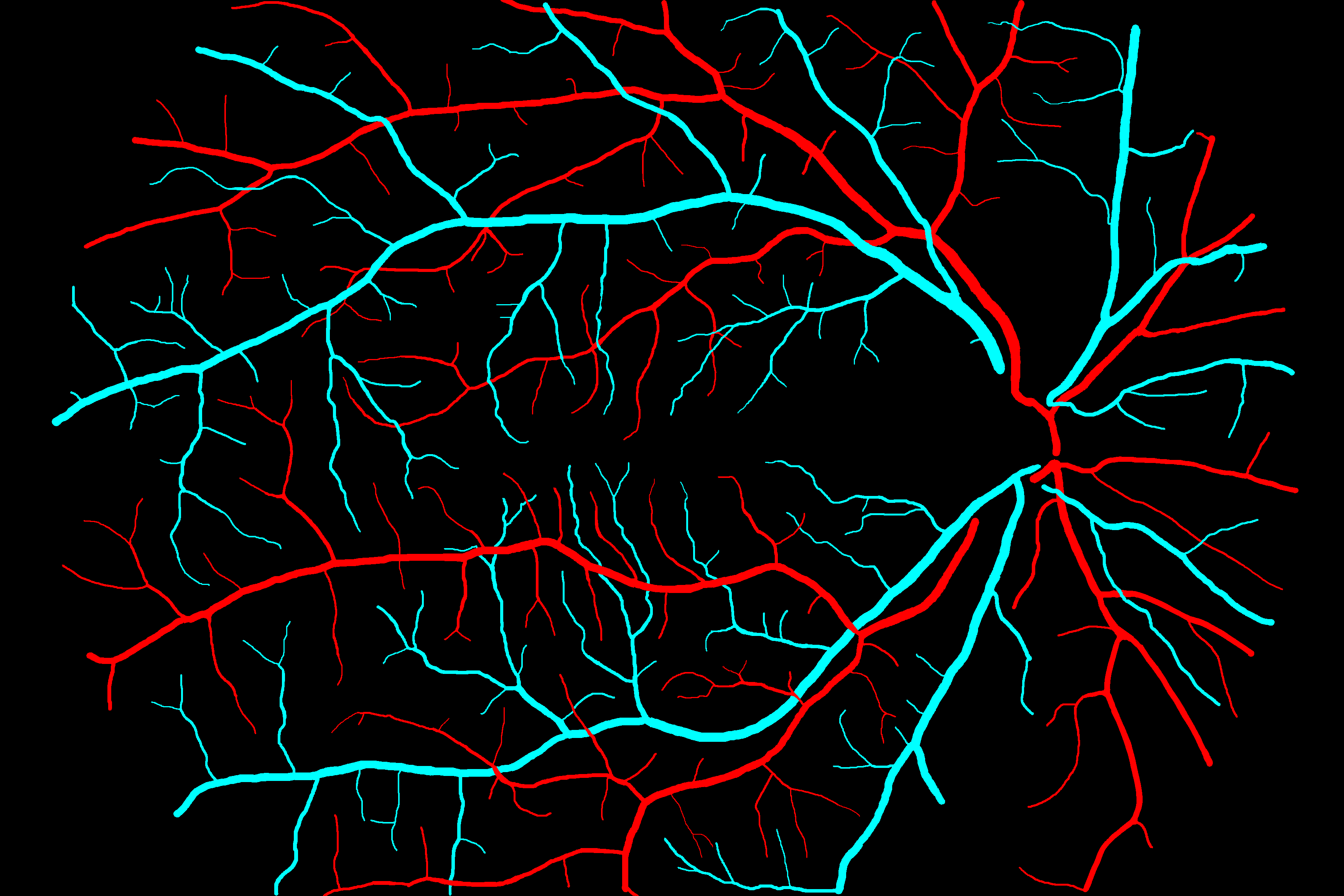

Supplement: Supplementary file 3 — Supplementary information [file 41598_2019_51850_MOESM3_ESM.zip › SLO/vessels_01_h_VeinArtery1.png]

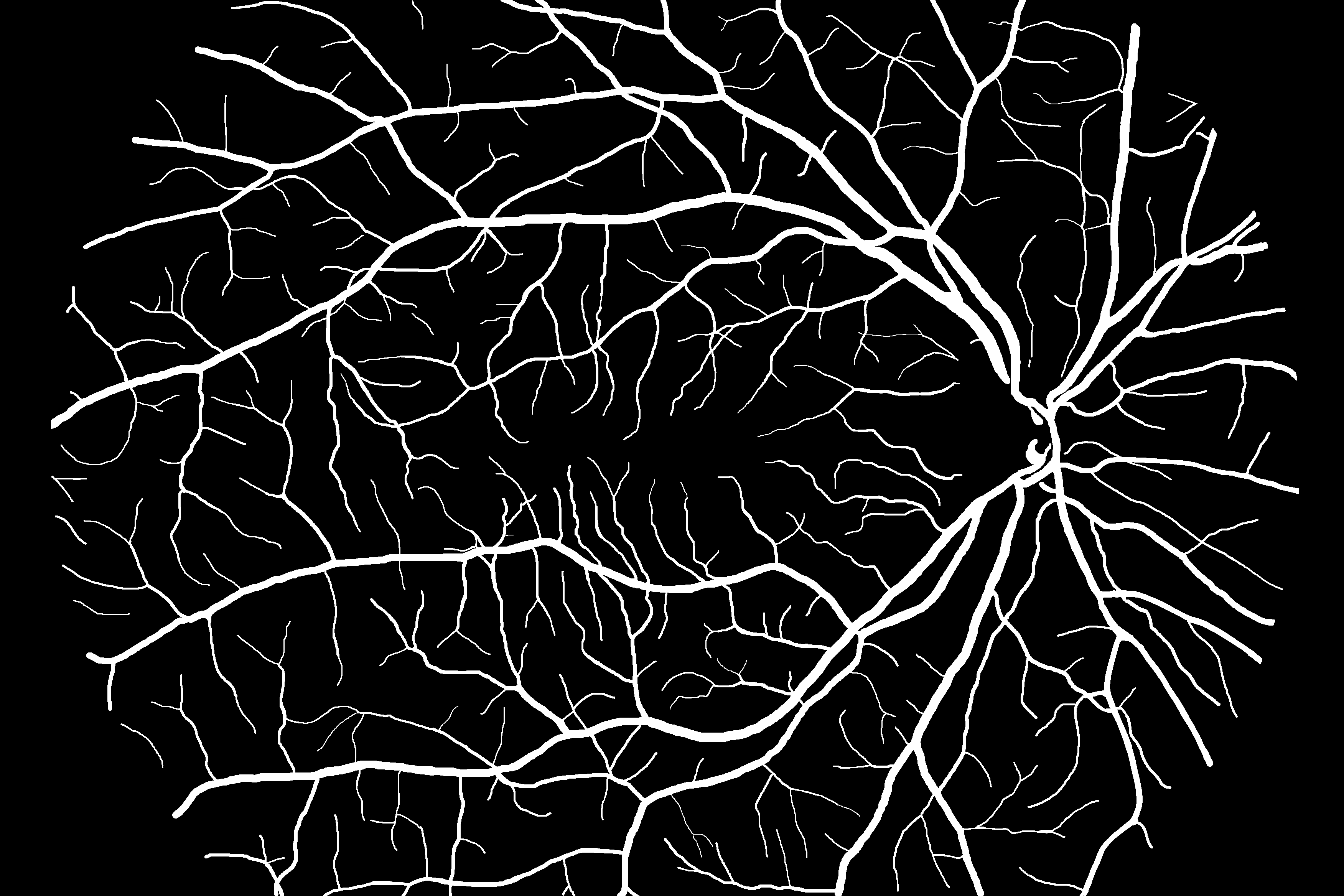

Supplement: Supplementary file 3 — Supplementary information [file 41598_2019_51850_MOESM3_ESM.zip › SLO/vessels_01_h_track.tif]

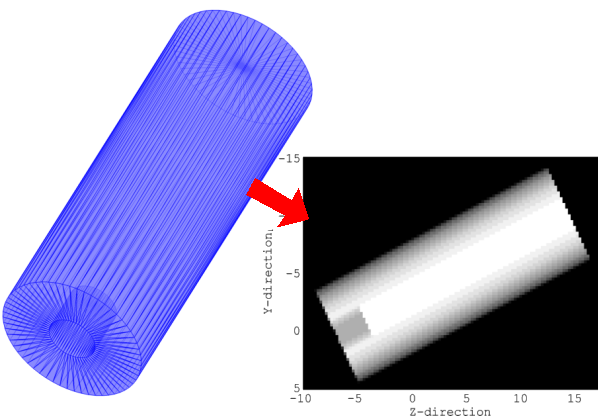

Supplement: Supplementary file 5 — Supplementary information [file 41598_2019_51850_MOESM5_ESM.zip › Matlab/Mesh_voxelisation/STLvoxelisation.png]
