# Supplementary material for: Holistic Monte-Carlo optical modelling of biological imaging
Source: Sci Rep. 2019 Nov 1;9:15832. doi: 10.1038/s41598-019-51850-1 (PMC6825179; doi:10.1038/s41598-019-51850-1)
Supplement: Supplementary file 4 — Supplementary information [file 41598_2019_51850_MOESM4_ESM.zip › readme_v4.pdf]

# Ray Tracing Polarisation-sensitive Mie Scattering for Zemax-OpticStudio™

*Version v4 (27 April 2017)*

This program calculates the parameters associated to a scattering event on performing Monte Carlo simulation of Mie scattering. For an incoming optical ray, it provides the location of the scattering event, the new direction of propagation and the electric field of the scattered ray. Additionally, fluorescence can be simulated by implementing a wavelength shift occurring randomly at a scattering event, and is modelled as isotropic incoherent emission. (Note: this version does not update the phase function after a wavelength shift.) A fluorescence mean-free path can be defined which can be set lower than the scattering mean-free path.

**Installation:**
Installation on a pre-installed Zemax-OpticStudio only requires extracting and copying files 'MSP_v4.dll' and 'MSP_v4.def' into folder '<installation directory>\DLL\BulkScatter'

**Usage:**
Navigate to the "Volume physics" tab on a volume-object defined in a non-sequential Zemax model. Select "DLL Defined Scattering" and select "MSP_v4" from the drop-down list. The material (glass or media) used within Zemax must not have absorption (if Lamber-Beer absorption is to be accounted 'Bulk absorption' parameter of this DLL should be used instead). The interface for entering scattering parameters is shown in Figure 1 and includes the following parameters:

| | |
|---|---|
| Mean Path | Mean-free path of light propagating in the medium in mm (the inverse of the scattering coefficient). |
| Transmission | Attenuation factor occurring at each scattering event. |
| Particle radius | Radius of the scattering particles |
| Index of refraction (real) | Real part of the index of refraction of the particles relative to the medium. |
| Index of refraction (imag) | Imaginary part of the index of refraction of the particles relative to the medium. If parameter 'Density' is not zero, imaginary part of the index of refraction will account for bulk absorption according to the absorption cross-section from Mie calculations; if parameter 'Density' is zero, the value is ignored. |
| Density | The density or concentration of scattering particles. If not zero, mean-free path is calculated and input from 'Mean Path' is ignored. |
| Bulk absorption | Lambert-Beer absorption of the medium. The material properties of the object must not have absorption defined and instead it should be accounted through this parameter. |
| Quantum efficiency | Attenuation of the intensity of the rays when fluorescence occurs. |
| Fluorescence mean-free path | Mean path travelled by rays before undergoing fluorescence at set probability. If set at zero, the value 'Mean path' will be used. If set higher that 'Mean Path', the value 'Mean Path' will be used. |

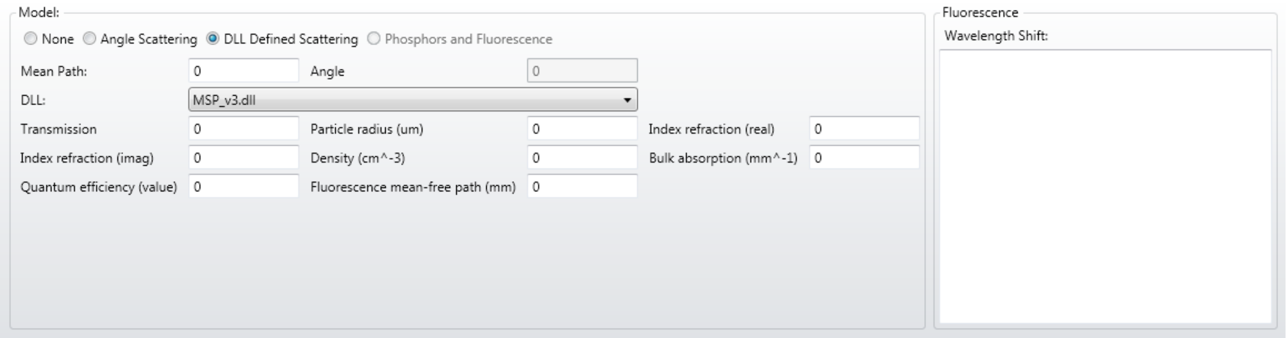


**Figure 1:** Interface of the DLL in Zemax-OpticStudio

**Notes:**

- MSP requires calculation of initial look up tables that is performed only once when the DLL is loaded into memory, and it is done on the launch of the first ray. This means there is a delay on tracing the first ray after loading the DLL or changing relevant parameters (particle size or particle refractive index). The table must be re-computed if the wavelength is changed.
- The DLL requires enough memory to store the tables.
- MSP can use the multiple CPUs with a single, shared, calculation of the look up tables. This enables fast raytracing.
- Only one object in the Zemax-OpticStudio model file can have MSP scattering enabled, if more than one object should have MSP scattering this can done by creating a copy of the MSP.dll file with a different name (for example ‘MSP_v4.dll’ and ‘MSP_v4copy.dll’ under folder ‘<zemax installation directory>\DLL\BulkScatter’), which enables to calculate one look up table per DLL copy.
- MSP detects fluorescence when observes a ray that has a different wavelength than the very first ray that is encounters. Therefore, in the current version:
  - Excitation rays are assumed to have a single wavelength
  - It is assumed that the first ray is at excitation wavelength
  - If the scattering object is hit by a ray with an emission wavelength, it will wrongly assume a fluorescence event at the surface of the object. To avoid this, and enable MSP for other objects, the program is also provided with a fluorescence-disabled DLL, ‘MSP_v4_nonfluor.DLL’

**Acknowledgements:**
This program incorporates Mie calculations performed using code described in: W. J, Wiscombe “Mie scattering calculations: Advances in technique and fast, vector-speed computer codes,” Tech. Rep. NCAR (1979); and in: W. J. Wiscombe “Improved mie scattering algorithms” Applied Optics, 19 (1980).
