# Supplementary material for: Holistic Monte-Carlo optical modelling of biological imaging
Source: Sci Rep. 2019 Nov 1;9:15832. doi: 10.1038/s41598-019-51850-1 (PMC6825179; doi:10.1038/s41598-019-51850-1)
Supplement: Supplementary file 5 — Supplementary information [file 41598_2019_51850_MOESM5_ESM.zip › AngularSpectrumCalculator/readme_AngSpecCalc.pdf]

Angular Spectrum sampler for Zemax-OpticStudio™

Version v1 (26 April 2017)

This program calculates the Angular Spectrum representation of a beam that is sampled by rays. The program is defined as a surface scattering DLL that shall be applied on a surface in Zemax-OpticStudio™ ; however the program does not scatter or alter the rays in any way. When a ray hits the surface the program stores its information in memory by converting the ray into a associated plane wave (with infinite extent). Upon performing a raytrace, when the specified number of rays have been accumulated, the program calculates the electric field at a grid on the plane of the surface and stores the output to disk, and saves the following two output files to disk:

- **C:\AngSpectrumCalc_output\EfieldGrid.txt** contains the value of the electric field values at the specified grid locations, see below for example of the format.
- **C:\AngSpectrumCalc_output\AngSpectrumComponents.txt** contains a list of the Angular Spectrum components (plane waves) that sampled the beam, see below for example of the format.

Installation:
Installation on a pre-installed Zemax-OpticStudio only requires extracting and copying file ‘AngSpectrumCalc_v1.dll’ and ‘AngSpectrumCalc_v1.def’ into folder ‘<installation directory>\DLL\SurfaceScatter’

Usage:
This program is to be used as a surface scattering. It is recommended to be used on a “Rectangle” object type. Navigate to “Coat/Scatter” on the Object Properties tab, and on the “Scatter” box select “User defined” for the drop-down list of the “Scatter model” property, and select “ AngSpectrumCalc_v1”, see screenshot below. Leave “Number Of Rays” and “Scatter Fraction” to 1. The size of the object (of the “Rectangle” object) determines what rays will be used for the calculation of the Angular Spectrum (as only rays that hit the surface will be considered) and this is determined by the “X Half Width” and “Y Half Width” parameters of the object (note this are different from the half-width parameters of the DLL). Additional parameters are:

**Num rays**	The number of rays to be accumulated before the electric field is calculated
**x half-width**	Half width in x of the grid where the electric field will be calculated
**y half-width**	Half width in y of the grid where the electric field will be calculated
**Nx**	Number of samples of the electric field grid in x
**Ny**	Number of samples of the electric field grid in y

Example usage:

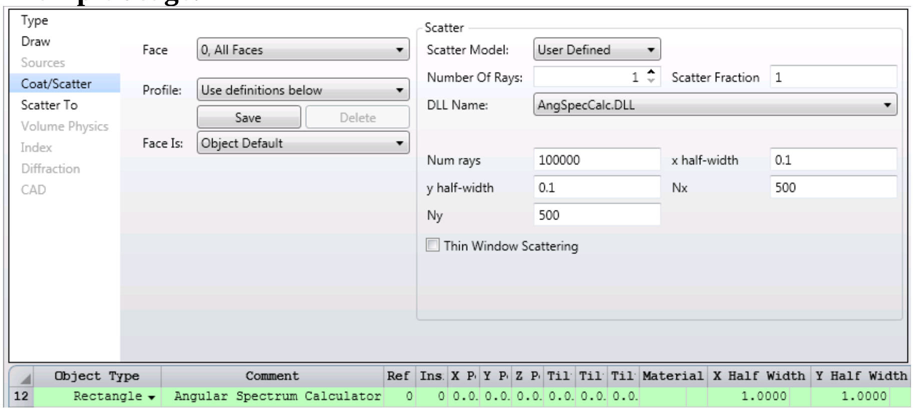

Notes:
It is recommended that more than “Num rays” are traced because the rays that for any reason miss the object will not contribute to sample the beam, and the program uses multi-thread calculations which require more rays to be launched. As a hint: ensure for example that “Num rays” + 500 rays hit the surface.

The program clears its internal memory when “Num rays” have been accumulated and the output files are generated, and will start accumulating rays again. To ensure a fresh start, user should reload the DLL or simply close and reopen Zemax.

Example format of the output files:

“C:\AngSpectrumCalc_output\EfieldGrid.txt”:

- The first line is: the number of rays that were used, and then the x locations of the grid (with zeros inserted between in each location)
- The left-most column (i.e. every first entry of each line) starting from the second line are the y location of the grid.
- Each other location is the value of the complex electric field in pairs of real value and imaginary value”
- Example:

```
100000; -3.000000000000e-002; 0; -2.970000000000e-002; 0; ...
-3.000000000000e-002;  1.152358943620e-001;  3.964609315995e-002;  1.142701532456e-001;  3.868756397464e-002; …
-2.994000000000e-002;  3.740591170098e-002;  7.102593121451e-002;  3.641409166151e-002;  7.002619598396e-002; …
-2.988000000000e-002; -4.470630727327e-002;  1.002497468834e-001; -4.572472570240e-002;  9.921023153327e-002; …
…
```

This example corresponds to:

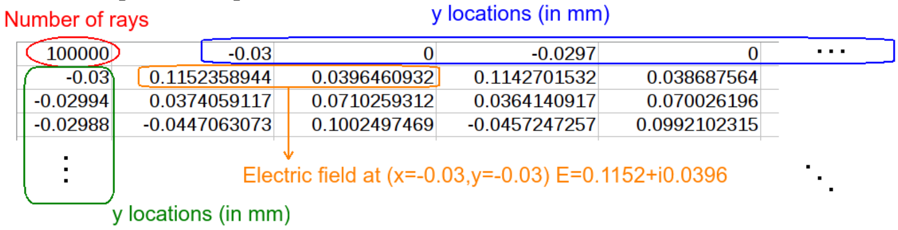

“C:\AngSpectrumCalc_output\AngSpectrumComponents.txt”

- The first line is: number of rays used, wavelength, index of refraction of the incident media, zero.
- Subsequent lines contain for each ray/plane-wave: kx, ky, phase, intensity
- Example:

```
100000;  4.880000000000e-001;  1.000000000000e+000; 0
 5.390812884489e-004;  1.008328812319e-001; -7.150567824307e+000;  4.472556410920e-006
-2.837412839111e-004; -1.282309406004e-001; -7.063344664625e+000;  4.430057541697e-006
 1.740539191926e-004;  6.713105017248e-002; -4.239714367205e+000;  4.491747351998e-006
-9.092213485555e-005;  7.222218681578e-002; -4.050105019228e+000;  4.484405158476e-006
```
