# Supplementary material for: Holistic Monte-Carlo optical modelling of biological imaging
Source: Sci Rep. 2019 Nov 1;9:15832. doi: 10.1038/s41598-019-51850-1 (PMC6825179; doi:10.1038/s41598-019-51850-1)
Supplement: Supplementary file 5 — Supplementary information [file 41598_2019_51850_MOESM5_ESM.zip › SourceFromDB/readme_SourceFromDB.pdf]

Light source from voxelised pattern for Zemax-OpticStudio™

Version v1 (26 April 2017)

This program generates light rays to be inserted in Zemax-OpticStudio™ as a light source in non-sequential mode. The program loads a table from disk that shall be generated in advance, and must contain a sequence of 3D coordinates and associated cumulative weights. The first line of the table contains the number of stored voxels and the intensity of each ray traced from the light source. Subsequent lines store 3D coordinates and associated cumulative weights. The probability that a ray is generated in each voxel is proportional to its increase in cumulative-weight with respect to the previous voxel (from the previous line). Cumulative weights shall be normalised such that cumulative weight of the last stored voxel is 1. The table shall be stored in local disk under the following path and name:

‘C:\SourceFromDB\intensity_map_num<num>.txt’

where <num> is an integer with 5 digits, for example ‘00001’. Additionally, the user may define the volume associated with each voxel, such that the origin of each ray is then generated in a random location with the volume.

Installation:
Installation on a pre-installed Zemax-OpticStudio only requires extracting and copying file ‘SourceFromDB_v1.dll’ and ‘SourceFromDB_v1 .def’ into folder ‘<installation directory>\DLL\Sources’

Usage:
This program is to be used through a ‘Source dll’ type from the available non-sequential light sources. Select ‘SourceFromDB_v1’ in the Data File drop-down menu under the General tab. Position and orientation of the light source may be used to define shifts and rotations with respect the the coordinates defined and stored in the table. Common parameters with all sources types are used except for the “Power” parameter, which is ignored as the intensity of each ray is set by the scale parameter defined in the first entry of the table. Additional parameters are:

num	Integer number used to indicate what input file to load
voxel x	Size of the voxels in x (in mm) – optional
voxel y	Size of the voxels in y (in mm) – optional
voxel z	Size of the voxels in z (in mm) – optional

Example usage:

Object Type	Comment	Ref Object	Inside Of	X Position	Y Position	Z Position	Tilt About X	Tilt About Y	Tilt About Z	Material	# Layout Ray:	# Analysis Ra	Power(Watts)	Wavenumber	Color #	num	voxel x	voxel y	voxel z
Source DLL ▾	SourceFromDB.dll	1	0	0.0000	0.0000	0.0000	0.0000	0.0000	0.0000	-	50	100000000	1.0000	2	0	300.0000	1.0000E-004	1.0000E-004	1.0000E-004

Example table content:
```
   6218 1.302560432775e+05
-0.0172500000 -0.0144500000  0.0010000000  0.00008034656002642
-0.0171500000 -0.0144500000  0.0010000000  0.00016069657478699
-0.0170500000 -0.0144500000  0.0010000000  0.00024105004428170
…
```
